# Implementation of a Hospital Medicine Rotation and Curriculum for Internal Medicine Residents

**DOI:** 10.15766/mep_2374-8265.10977

**Published:** 2020-09-29

**Authors:** Doris Lin, Chirayu Shah, Erica Lescinskas, Cory Ritter, Lindsey Gay

**Affiliations:** 1 Assistant Professor, Department of Medicine, Baylor College of Medicine; 2 Associate Professor, Department of Medicine, Baylor College of Medicine; Associate Program Director, Internal Medicine Residency, Baylor College of Medicine; 3 Assistant Professor, Department of Medicine, Baylor College of Medicine and Michael E. DeBakey VA Medical Center

**Keywords:** Hospital Medicine Rotation and Curriculum, Hospital Medicine, Internal Medicine, Curriculum Development

## Abstract

**Introduction:**

Hospital medicine is a growing field that focuses not only on expertise in inpatient medicine but also on knowledge of nonclinical health system topics. The traditional model for resident education does not lend itself to learning these topics. We developed a unique ward rotation with a dedicated curriculum called the Resident Inpatient Training Experience (RITE) to address this deficiency.

**Methods:**

The RITE rotation was initially implemented in the 2013–2014 academic year. The curriculum accompanying the rotation contained four case-based modules that included content on patient safety, quality improvement, cost-conscious care, hospital metrics/reimbursement, physician billing and coding, and transitions of care. Prior to RITE, residents received an email orientation to the service. To evaluate the rotation and curriculum, residents completed a pre- and postrotation online survey. Forty-six upper PGY 2 residents each rotated on the service for 1 month. An experienced hospitalist attended on the service and facilitated a weekly discussion on each module. This publication includes an updated version of the email orientation, the four modules, and the surveys.

**Results:**

There was a 72% response rate for completion of the pre- and postrotation survey. Confidence in managing hospitalized patients and knowledge of module content taught during the rotation improved.

**Discussion:**

We found that implementation of a hospital medicine rotation and curriculum improved resident independence and knowledge of the module topics and was a successful way to alleviate current deficiencies in resident education.

## Educational Objectives

At the end of this rotation, learners will be able to:

1. Illustrate the framework for patient safety and the quality improvement process.
2. Demonstrate cost-conscious care and methods to improve hospital metrics and reimbursement.
3. Select the appropriate inpatient physician billing level based on documentation.
4. Examine current transitions-of-care models and determine causes of ineffective transitions.
5. Select the appropriate postdischarge placement for patients.

## Introduction

Originating in the 1990s, hospital medicine is a rapidly expanding field, with over 50,000 practicing hospitalists in 2016.^1^ Not only do current hospitalists manage hospitalized patients, their role has expanded to include involvement in teaching, research, comanagement, and leadership.^2–5^ As the field continues to grow as a subspecialty of internal medicine, there remains a training gap in residency programs in medicine topics that fall outside traditional curricula. At our institution, we recognized the need for and importance of formal education on these topics as well as improved independence in managing patients throughout an entire hospital course that was occurring less often on the traditional wards rotation. In addition, the implementation of Accreditation Council for Graduate Medical Education duty-hour restrictions has limited independence in inpatient responsibilities as residents now receive an increased number of handoffs and often do not care for a patient throughout the entire hospital course.^6–8^ There is also less time for additional didactics on these important topics.

Previous studies have supported the need to create hospitalist-focused training in quality improvement (QI), patient safety, health care systems, and health care economics.^9^ In 2006, the Society of Hospital Medicine published a blueprint for core competencies in hospital medicine consisting of three main categories: clinical conditions, procedures, and health care systems.^10^ The blueprint highlighted the role of hospitalists not only as clinicians but also as leaders in improving the hospital system in which they work. Our workbook curriculum focused on enhancing resident education in health care system areas including QI, patient safety, cost-conscious care, hospital metrics/reimbursement, physician billing, and transitions of care.

Modules and curricula on QI and patient safety^11–13^ and a few on coding and documentation^14,15^ can be found in *MedEdPORTAL*. However, they are all separate curricula and do not include physician billing and transitions of care. These prior publications also do not incorporate their curricula into an entirely new and integrated hospital medicine rotation specifically for internal medicine residents. In addition, there are few evidence-based curricular resources that measure outcomes of educating residents on the specific topics that have been highlighted as significant health care system areas. With the increasing popularity of hospital medicine as a career choice for graduating internal medicine residents, it is imperative that they be introduced to these topics during residency. Therefore, we have developed and implemented a rotation with a comprehensive curriculum encompassing hospital medicine topics that we call the Resident Inpatient Training Experience (RITE).

The overall goal has been to make this curriculum available for educators in internal medicine and other specialties where these topics would be pertinent to learner education. The topics are applicable to all health care systems and can be easily implemented in any graduate medical education program.

## Methods

### Development of the Rotation

In order to mirror a true hospitalist experience, only upper-level residents rotated on the service, without interns and medical students. The rotation was initially implemented in the 2013–2014 academic year and has been an active service at two affiliated hospitals since then. Over the last year, the rotation was also added to a third affiliated hospital. We selected hospitalists to attend on the RITE team based on experience and interest in resident education and oriented them to the purpose and structure. Hospitalists were given a workbook to become familiar with the module topics they would be responsible for teaching. The workbook curriculum served as the template and formal guide for the facilitators. Future facilitators can also easily utilize the guided facilitated discussion detailed below for each module.

A few days prior to the beginning of the rotation, each resident received an email orientation to the service. The orientation included a brief description and introduction to the service (Appendix A). It outlined the structure, patient care responsibilities, and expectations. It also introduced the printed workbook curriculum and instructed the residents to complete the online pre- and postrotation survey (Appendices B and C). The email orientation can be easily edited to fit the structure and call schedule of individual residency programs.

Two PGY 2 residents each rotated on the service for 1 month. The rotation was set up at two affiliated sites: (1) a 486-bed tertiary care county hospital and level I trauma center and (2) a Veterans Affairs hospital that included 357 acute care beds, a 40-bed spinal cord injury center, and a 141-bed community living center. At each site, the team had its own team room and admitted up to four patients per day between 7:00 a.m. and 5:00 p.m., 4 days a week. The hospitalist attending conducted daily rounds and encouraged development of individual assessment and plans. The residents were responsible for fielding pages, charting admissions and discharges, writing daily notes, and working in a multidisciplinary fashion with other services. Each resident managed his/her own set of patients throughout the hospital course and coordinated care with ancillary staff, including nurses, case managers, social workers, and consultants. There was no overnight call; the residents verbally signed out the patients to the covering resident in the evening and received a verbal sign-out in the morning. One resident covered all the patients on the weekends with attending oversight.

Each week, the residents completed a module from the workbook curriculum in advance of the facilitated discussion. The discussion typically took place on the afternoon of the nonadmitting day, and the residents were encouraged to ask questions and apply the new knowledge to their daily workflow and clinical practice. Although each facilitated discussion lasted approximately 1 hour, the actual application of the module concepts was emphasized during daily rounds in order to optimize the learning experience.

### Development of the Curriculum

We decided on the topics to be covered in each module through a review of the literature and consensus among a group of hospitalists. The printed workbook curriculum contained four modules that focused on improving knowledge of specific health care system topics, including patient safety, QI, hospital metrics/reimbursement, cost-conscious care, physician billing and coding, and transitions of care. Some of the module topics had been previously identified as core competencies in hospital medicine^10^ and were also identified by us as topics deficient in our program.

#### Module 1

Basic Principles in Patient Safety (Appendix D) described the extent and cost of medical errors in the United States. It also defined and categorized medical errors and gave examples of the types of errors physicians see in the clinical setting.

#### Module 2

Quality Improvement, Hospital Metrics/Reimbursement, and Cost-Conscious Care (Appendix E) described the Institute of Medicine's six aims for QI^16^ and reviewed the design process of a QI project using the plan-do-study-act (PDSA) model. The module also described common performance and quality metrics currently measured by hospitals and concluded with a summary of cost-conscious care.

#### Module 3

Physician Billing and Coding (Appendix F) provided a basic introduction to the documentation necessary in an initial and a subsequent inpatient hospital care note in order to support the three levels of physician billing and defined relative value units.

#### Module 4

Transitions of Care (Appendix G) reviewed several evidence-based models that identified obstacles that could arise in the discharge process. It also reviewed strategies to develop hospital QI initiatives. The module ended with a review of the necessary criteria to qualify for different postacute care facilities.

#### Updates to modules

The curriculum has been updated since inception based on feedback and changes in program curricula. However, the core topics have remained relatively unchanged. The protocol was approved by the institutional review board.

### Development of the Survey

We performed an assessment of resident confidence in managing patients, knowledge of module topics, and interest level before and after the rotation through a pre- and postrotation online survey. Questions were designed by author consensus. The confidence level and the residents' view of hospital medicine statements in both surveys were based on a 5-point Likert scale (1 = *strongly disagree,* 5 = *strongly agree*). Residents' knowledge level of hospital medicine topics was based on a 5-point Likert scale (1 = *very poor,* 5 = *excellent*) in both surveys.

### Module 1: Basic Principles in Patient Safety

This module introduced learners to the concepts of patient safety (Appendix D). It began with a prelecture assignment and was followed by the clinical scenario of an admitted elderly patient with cellulitis who was faced with several safety challenges that arose during her hospitalization. The instructor facilitated a discussion with the residents of personal experiences with patient safety issues they had faced working in their own hospital system (10 minutes). This was followed by an introduction on the extent and cost of medical errors in the United States and the Swiss cheese model of weaknesses in a complex medical system (10 minutes).^17^ Next, the instructor returned to the initial clinical scenario and discussed the holes that had occurred in the patient's care (10 minutes). The module also defined medical error and its subdivision into near misses and adverse events. It described types of adverse events and gave examples of interventions that could be implemented to reduce errors. The module continued by describing Leape, Lawthers, Brennan, and Johnson's characterization of errors into four categories: diagnostic, treatment, preventive, and others.^18^ The resident selected the category of error for each practice case (10 minutes). The module ended with a facilitated exercise on the original clinical scenario where the resident identified and characterized the medical errors and provided suggestions for prevention of these errors in the future (15 minutes).

### Module 2: Quality Improvement, Hospital Metrics/Reimbursement, and Cost-Conscious Care

The concepts of QI, hospital metrics, inpatient reimbursement, and cost-conscious care were reviewed in this module (Appendix E). The module began with an introduction to the Institute of Medicine's aims for QI^16^ and the Institute of Healthcare Improvement's model^19^ for improvement as a framework for implementing QI projects. This was followed by a case presentation of low-value imaging for patients at low risk of having a pulmonary embolism. The facilitator led the trainees through the process of developing a PDSA cycle through guided open-ended questions (15 minutes). Next, the focus shifted to utilization management and determination of appropriate levels of care in hospitalized patients. The facilitator provided background on commercial projects used to determine appropriate levels of care and how this could affect hospitals. Residents completed a case-based exercise where they were asked to determine the appropriate level of care based on a patient's presenting history, exam, and initial diagnostic workup (10 minutes).

In its next section, the module introduced learners to the inpatient prospective payment system and the basic framework through which hospitalizations could be characterized for determination of case weight, expected length of stay, and reimbursement. Instructors led the residents through an exercise where they reviewed their own documentation on current patients to identify opportunities for documentation optimization (15 minutes). Next, the instructor introduced residents to Centers for Medicare and Medicaid Services programs aimed to improve care quality by performance-based adjustments to hospital reimbursement. These programs included value-based purchasing, readmission reduction, and health care–associated complications reduction (10 minutes). Lastly, the module ended with an introduction to cost-conscious care. Residents estimated charges of frequently ordered inpatient studies. The facilitator then provided them with the actual charge amounts for these studies. At the end, the residents analyzed studies ordered on patients under their care and determined which tests were likely unnecessary (10 minutes).

### Module 3: Physician Billing and Coding

The third module introduced learners to the documentation necessary in an initial and a subsequent inpatient care note in order to support the three levels of physician billing (Appendix F). As a prelecture assignment, learners reviewed two of their own history and physical exam (H&P) notes and assigned an arbitrary code of low, moderate, or high complexity. The learners also assigned an arbitrary code to the sample H&P documented in the module. Next, the instructor reviewed the three levels of billing and discussed in detail the components necessary to bill each level, which were organized into charts in the workbook (20 minutes). After discussing each billing level, the instructor returned to the sample H&P and performed a step-by-step discussion with the learners to decide what billing level would be appropriate (10 minutes). The instructor then reviewed the definition of relative value units, associated work relative value units, and reimbursement assigned to each level of billing (5 minutes). The instructor repeated the steps with the residents' own H&P and subsequent day note utilizing the billing charts provided in the workbook to decide what level was met (20 minutes). Lastly, the instructor emphasized the medical decision-making component to avoid overbilling.

### Module 4: Transitions of Care

Residents learned the importance of appropriately transitioning patients from the inpatient to the outpatient setting (Appendix G). The module began with a clinical scenario outlining a patient who had been discharged on warfarin and was subsequently readmitted with an intracerebral hemorrhage after lack of follow-up. This introduced the importance of preventable readmissions and their effects on hospital reimbursement (5 minutes). The instructor introduced several evidence-based transitions-of-care models that had been previously developed to improve patient outcomes, including Better Outcomes for Older Adults through Safe Transitions from the Society of Hospital Medicine,^20^ Project RED (Re-Engineered Discharge) from Boston Medical Center,^21^ and others referenced in the module as optional reading. The instructor discussed the different obstacles to safe transitions identified in these resources and summarized in the module as areas for improvement (15 minutes). The instructor engaged the learners to discuss areas that could be improved in discharging patients at their own institution and highlighted their past experiences with difficult patient transitions (15 minutes). The instructor then returned to the initial clinical scenario and facilitated a discussion identifying the changes that could have been made to prevent the patient's readmission (10 minutes). Lastly, the instructor reviewed the different types of postacute care settings and the criteria for admission into each setting (10 minutes).

### Evaluation of the RITE Rotation and Curriculum

Prior to the beginning and at the end of the rotation, the residents were asked to complete an online survey (Appendices B and C). The data from the pre- and post-RITE surveys were analyzed using the Wilcoxon signed rank test. Only the residents who completed both surveys were included in the analysis.

## Results

**Figure 1.**
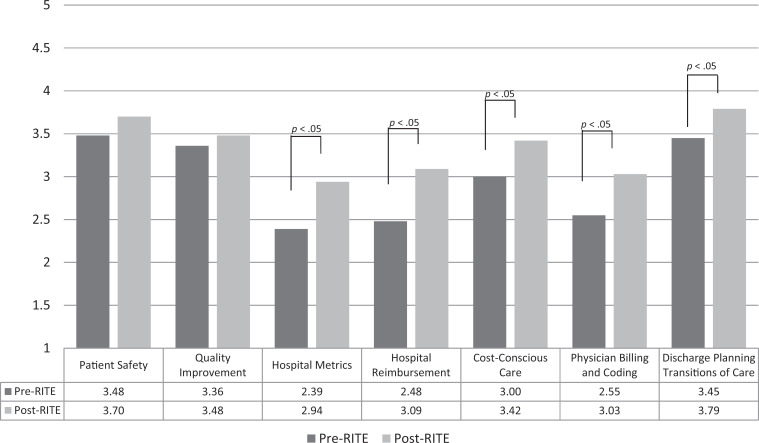
Residents' mean knowledge level of hospital medicine topics before and after rotation (*n* = 33). Rated on a 5-point Likert scale (1 = *very poor*, 5 = *excellent*). Abbreviation: RITE, Resident Inpatient Training Experience.

Since July 2013, over 150 PGY 2 residents have completed the rotation. The data on the confidence, knowledge, and perceived deficits in training questions have been published previously.^22^ A total of 33 PGY 2 residents (response rate of 72%) completed the pre- and postrotation surveys from July 2013 to June 2014. These responses were analyzed using the Wilcoxon signed rank test. Prerotation survey results indicated resident-reported training deficiencies in all of the module topics, with the most deficits in hospital reimbursement (79%), hospital metrics (61%), physician billing and coding (58%), cost-conscious care (58%), and transitions of care (28%). After completing the rotation, residents' self-assessed knowledge level of hospital reimbursement, hospital metrics, physician billing and coding, cost-conscious care, and transitions of care rose significantly (Figure 1). Knowledge level of QI and patient safety did not improve significantly, which we attributed to the implementation of a separate QI and patient safety curriculum for the residents.

**Figure 2.**
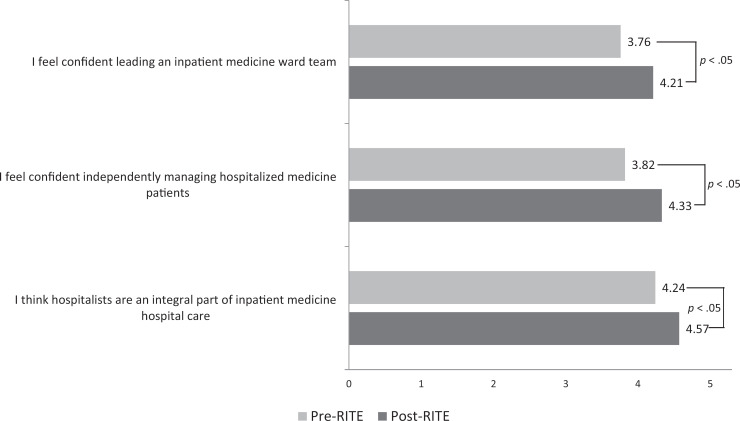
Resident mean confidence level and understanding of hospitalist role (*n* = 33). Rated on a 5-point Likert scale (1 = *strongly disagree*, 5 = *strongly agree*). Abbreviation: RITE, Resident Inpatient Training Experience.

Residents' understanding that hospitalists serve as an integral part of inpatient care and their confidence in independently managing hospitalized medical patients also improved significantly (Figure 2). In addition, 100% of the residents either agreed or strongly agreed that the rotation would help them as they transitioned to upper levels in order to successfully lead a medicine ward team. After completion of the RITE service, many residents commented that they enjoyed the “high level of autonomy” and were given “more independence in patient care decisions than on the wards month.” Other residents felt it “solidified” what they learned during intern year and gave them “confidence to manage patients” on their own. Approximately 66% of the residents also reported that using the workbook modules and facilitated discussion with an experienced hospitalist was an effective method of delivering the curriculum (Table). Lastly, interest level in hospital medicine improved, and residents cited job satisfaction, work-life balance, and ease of finding a job as reasons for this elevated interest.

**Table.**
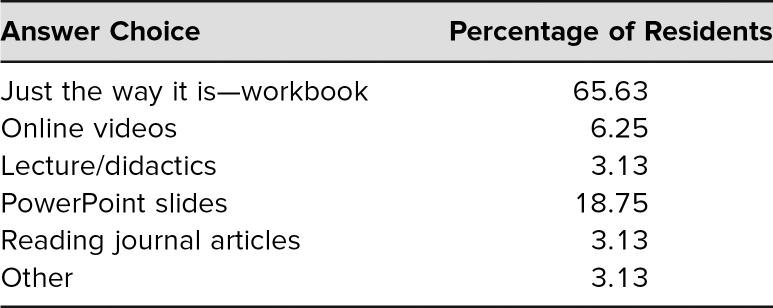
Resident Preference on Curriculum Delivery Methods (*N* = 33)

## Discussion

We created a unique rotation with its own dedicated curriculum to fill the gap in trainee knowledge of nonclinical medicine topics and hospitalist-focused training. The rotation provided residents with much-needed experience in managing patients throughout an entire hospital course, and the curriculum coupled with facilitated discussion led by an experienced hospitalist significantly improved knowledge of hospital metrics/reimbursement, physician billing and coding, cost-conscious care, and transitions of care.

Since knowledge of these topics is important in almost all medical fields, it made us review our current teaching in the internal medicine residency program. Prior to this rotation, the residents may have received didactics in QI and patient safety, but very little (if any) education on the other topics. We also realized that adding these lectures to an already compressed conference schedule was not feasible. Our rotation and curriculum improved many aspects of trainee skill and knowledge over a 1-month time period.

Before the rotation, the survey indicated that 95% of the respondents agreed or strongly agreed that this type of curriculum would be important for their residency training. Further review of the postrotation survey and comments indicated that the residents enjoyed the autonomy of managing their own set of patients without the additional responsibility of supervising interns and medical students. This provided great practice and preparation to build the skills they needed to lead a ward team. In addition, residents preferred hospitalists as attendings on medical ward teams. A prior meta-analysis demonstrated that trainees were more satisfied with teaching from hospitalists compared to nonhospitalists^23^ and were rated highly for teaching effectiveness.^24–27^ Furthermore, hospitalists are generally more available and have expertise in managing acute medical issues.

There were a few limitations to the study. First, the rotation and curriculum were implemented at two affiliated sites with two different hospitalist groups, so the teaching and overall experience may have varied depending on the attending on service. The curriculum was the same to provide consistency, and the residents were instructed to independently review each module prior to the facilitated discussion. Future instructors can utilize the guided facilitated module discussions detailed above. Second, our surveys assessed learners' perception of their own knowledge, which may not always have equated to actual knowledge. Adding a pre- and postrotation test on the module topics might provide a better assessment of learner knowledge improvement. In addition, adding a faculty postimplementation survey would be beneficial to assess whether the structure facilitates or limits teaching and learning on the RITE service. Future research directions could include adding these evaluations at the completion of the rotation. Lastly, incorporating facilitated discussions during a sometimes-busy clinical service was also a challenge. We tried to mitigate this challenge by having one weekday afternoon where the residents did not have to admit new patients, leaving that time devoted to education.

The goal of a curriculum such as ours is to improve confidence in managing patients and knowledge of important nonclinical topics. The robust modules provide up-to-date information along with realistic scenarios on patient care issues and daily challenges in health care systems. The case-based discussions offer an opportunity for high-level learning, analysis, synthesis, and evaluation. The curriculum can also be easily adapted to any graduate medical education program. Having a rotation specifically geared towards upper-level residents and experienced hospitalists also provides an excellent basis for piloting other inpatient initiatives and projects. In conclusion, the RITE rotation and curriculum constitute an innovative experience that can improve resident autonomy and introduce a novel approach to educating residents on important hospital medicine topics.

## Appendices

RITE Orientation Email.docxPre-RITE Survey.docxPost-RITE Survey.docxModule 1 Patient Safety.docxModule 2 QI, Metrics, Reimbursement, & Care.docxModule 3 Physician Billing & Coding.docxModule 4 Transitions of Care.docx
All appendices are peer reviewed as integral parts of the Original Publication.

## References

[1] Wachter RM, Goldman L Zero to 50,000—the 20th anniversary of the hospitalist. N Engl J Med. 2016;375(11):1009–1011. 10.1056/NEJMp1607958 27508924

[2] Pantilat S What is a hospitalist? Hospitalist. 2006;(2):123072 https://www.the-hospitalist.org/hospitalist/article/123072/what-hospitalist

[3] Pham HH, Devers KJ, Kuo S, Berenson R Health care market trends and the evolution of hospitalist use and roles. J Gen Intern Med. 2005;20(2):101–107. 10.1111/j.1525-1497.2005.40184.x 15836541 PMC1490059

[4] Lindenauer PK, Pantilat SZ, Katz PP, Wachter RM Hospitalists and the practice of inpatient medicine: results of a survey of the National Association of Inpatient Physicians. Ann Intern Med. 1999;130(4)(pt 2):343–349. 10.7326/0003-4819-130-4-199902161-00003 10068403

[5] Goldenberg J, Glasheen JJ Hospitalist educators: future of inpatient internal medicine training. Mt Sinai J Med. 2008;75(5):430–435. 10.1002/msj.20075 18828164

[6] Institute of Medicine. Resident Duty Hours: Enhancing Sleep, Supervision, and Safety. National Academies Press; 2009. 25009922

[7] Bump GM, Zimmer SM, McNeil MA, Elnicki DM Hold-over admissions: are they educational for resident? J Gen Intern Med. 2014;29(3):463–467. 10.1007/s11606-013-2667-y 24163152 PMC3930790

[8] Horwitz LI, Krumholz HM, Green ML, Huot SJ Transfers of patient care between house staff on internal medicine wards: a national survey. Arch Intern Med. 2006;166(11):1173–1177. 10.1001/archinte.166.11.1173 16772243

[9] Glasheen JJ, Siegal EM, Epstein K, Kutner J, Prochazka AV Fulfilling the promise of hospital medicine: tailoring internal medicine training to address hospitalists' needs. J Gen Int Med. 2008;23(7):1110 10.1007/s11606-008-0646-5 PMC2517911 18612754

[10] Dressler DD, Pistoria MJ, Budnitz TL, McKean SCW, Amin AN Core competencies in hospital medicine: development and methodology. J Hosp Med. 2006;1(1):48–56. 10.1002/jhm.6 17219471

[11] Werner JA An integrated, multimodal resident curriculum in patient safety and quality improvement. MedEdPORTAL. 2017;13:10641 10.15766/mep_2374-8265.10641 30800842 PMC6338155

[12] Keefer P, Orringer K, Vredeveld J, Warrier K, Burrows H Developing a quality improvement and patient safety toolbox: the curriculum. MedEdPORTAL. 2016;12:10385 10.15766/mep_2374-8265.10385

[13] Stewart D, Lye C, Lopez M, Mothner B, Camp E, Vachani J Engaging learners through modules in quality improvement and patient safety. MedEdPORTAL. 2016;12:10482 10.15766/mep_2374-8265.10482 30984824 PMC6440404

[14] Lee EL, Chisty A, Williams P, Brady W An innovative curriculum using a simulated electronic health record to teach internal medicine residents about ICD–10-CM. MedEdPORTAL. 2017;13:10538 10.15766/mep_2374-8265.10538 30800740 PMC6342159

[15] Chick D, Andreae M Diagnosis coding for clinicians: core knowledge and transition to ICD-10. MedEdPORTAL. 2014;10:9823 10.15766/mep_2374-8265.9823

[16] Institute of Medicine. Crossing the Quality Chasm: A New Health System for the 21st Century. National Academies Press; 2001. 25057539

[17] Reason J Human error: models and management. West J Med. 2000;172(6):393–396. 10.1136/ewjm.172.6.393 10854390 PMC1070929

[18] Leape LL, Lawthers AG, Brennan TA, Johnson WG Preventing medical injury. QRB Qual Rev Bull. 1993;19(5):144–149. 10.1016/S0097-5990(16)30608-X 8332330

[19] Varkey P, Reller MK, Resar RK Basics of quality improvement in health care. Mayo Clin Proc. 2007;82(6):735–739. 10.1016/S0025-6196(11)61194-4 17550754

[20] Hansen LO, Greenwald JL, Budnitz T, et al Project BOOST: effectiveness of a multihospital effort to reduce rehospitalization. J Hosp Med. 2013;8(8):421–427. 10.1002/jhm.2054 23873709

[21] Preventing avoidable readmissions: information and tools for clinicians—Project RED. Agency for Healthcare Research and Quality Accessed April 11, 2012. http://www.ahrq.gov/qual/impptdis.htm

[22] Lin D, Shah C, Campbell S, Bates JT, Lescinskas E Getting it RITE: impact of a dedicated hospital medicine curriculum for residents. South Med J. 2018;111(1):30–34. 10.14423/SMJ.0000000000000751 29298366

[23] Natarajan P, Ranji SR, Auerbach AD, Hauer KE Effect of hospitalist attending physicians on trainee educational experiences: a systematic review. J Hosp Med. 2009;4(8):490–498. 10.1002/jhm.537 19824099

[24] Chung P, Morrison J, Jin L, Levinson W, Humphrey H, Meltzer D Resident satisfaction on an academic hospitalist service: time to teach. Am J Med. 2002;112(7):597–601. 10.1016/S0002-9343(02)01155-5 12015263

[25] Hauer KE, Wachter RM, McCulloch CE, Woo GA, Auerbach AD Effects of hospitalist attending physicians on trainee satisfaction with teaching and with internal medicine rotations. Arch Intern Med. 2004;164(17):1866–1871. 10.1001/archinte.164.17.1866 15451761

[26] Geskey JM, Kees-Folts D Third-year medical students' evaluation of hospitalist and nonhospitalist faculty during the inpatient portion of their pediatrics clerkships. J Hosp Med. 2007;2(1):17–22. 10.1002/jhm.145 17274044

[27] Arora B, Wetterneck T, Schnipper J, et al The effects of hospitalist teaching attendings on medical student satisfaction and career interest: results from the multicenter hospitalist study. Presented at: Society of Hospital Medicine Annual Meeting; April 28–30, 2005; Chicago, IL.

