# Supplementary material for: Implementation of a Hospital Medicine Rotation and Curriculum for Internal Medicine Residents
Source: MedEdPORTAL. 2020 Sep 29;16:10977. doi: 10.15766/mep_2374-8265.10977 (PMC7526505; doi:10.15766/mep_2374-8265.10977)
Supplement: Supplementary file 1 — RITE Orientation Email.docxPre-RITE Survey.docxPost-RITE Survey.docxModule 1 Patient Safety.docxModule 2 QI, Metrics, Reimbursement, & Care.docxModule 3 Physician Billing & Coding.docxModule 4 Transitions of Care.docx [file mep_2374-8265.10977-s001.zip › A. RITE Orientation Email.docx]

**Welcome to the RITE Team**

The Resident Inpatient Training Experience (RITE) will introduce you to the practice of Hospital Medicine and help you improve your skills as an inpatient physician. This email contains information about the rotation structure and resident expectations.

**Introduction**The RITE service has been designed to provide residents with a unique hospital medicine experience.  The rotation includes active patient management, real-time use of evidence-based medicine, development of scholarly activities, and learning basic principles of Hospital Medicine.

**Pre- and Post-Rotation Survey**

Please complete the pre-rotation online survey a few days prior to beginning the rotation. Please complete the post-rotation online survey within a week of completing the rotation.

**Rotation Structure**The RITE service will consist of 2 upper level residents supervised by one hospitalist faculty member.  No interns or medical students are assigned to this rotation.  The admitting schedule is as follows with the admission end time of 5pm.  Monday-Thursday- 4 admissions/day, Friday-Sunday- no admissions.

Since there is no continuity clinic on this rotation, you should be able to devote more time towards your patients and your education.  A Workbook Module has been assigned each week.  All patients must be seen on the weekends.  Participating in Morning Report and noon conference is **mandatory** every weekday.  All off days should occur on the weekends.

**Patient Care**Residents are expected to arrive no later than 7am each morning to start evaluating current patients on the service.  The residents will provide a printed out checkout to the Night Float every day.  The checkout sheet should include your cell phone/home phone for the overnight resident to call for urgent issues.

All admissions will be called directly to the RITE pager.  This pager and the team phone should be passed between the residents so it is always answered.  Bedside rounding and teaching is the expectation.  In addition, please incorporate evidence-based medicine and what you learn in the weekly modules into your daily patient care.

Discharge summaries must be completed at discharge.  Please keep a list to follow up on patients that you have discharged.

At least one resident must cross cover all the patients during the weekends.  You can arrange the schedule between yourselves.

**Weekly Modules and Facilitated Discussion**A unique curriculum has been developed to discuss Hospital Medicine topics during this rotation.  Each resident will receive a printed workbook consisting of four modules: Module 1- Basic Principles in Patient Safety, Module 2- Quality Improvement, Hospital Metrics/Reimbursement, and Cost-Conscious Care, Module 3- Physician Billing and Coding, and Module 4- Transitions of Care.  Each resident is expected to read and complete each module **prior to** the workshop session.  Your attending will review the topics and facilitate a discussion on each module on your non-admitting day.

**Feedback/Evaluation**Please remind the attending to provide mid- and end of rotation feedback about your performance.  Part of your evaluation will include your completion of the readings/assignments and participation in the workshop sessions.

**Attending Specific Expectations**Please discuss any other specific expectations with the attending at the beginning of the rotation.

**And the last goal of the rotation…**
***Have fun!***
