# Supplementary material for: Implementation of a Hospital Medicine Rotation and Curriculum for Internal Medicine Residents
Source: MedEdPORTAL. 2020 Sep 29;16:10977. doi: 10.15766/mep_2374-8265.10977 (PMC7526505; doi:10.15766/mep_2374-8265.10977)
Supplement: Supplementary file 1 — RITE Orientation Email.docxPre-RITE Survey.docxPost-RITE Survey.docxModule 1 Patient Safety.docxModule 2 QI, Metrics, Reimbursement, & Care.docxModule 3 Physician Billing & Coding.docxModule 4 Transitions of Care.docx [file mep_2374-8265.10977-s001.zip › B. Pre-RITE Survey.docx]

**Pre-RITE Survey**

1. How many times have you led a ward team thus far?

- 0
- 1
- 2
- 3
- 4
- ≥ 5

2. Please choose you level of agreement with each of the following statements

|  | Strongly Disagree | Disagree | Neither Agree Nor Disagree | Agree | Strongly Agree |
| --- | --- | --- | --- | --- | --- |
| I feel confident independently managing hospitalized medicine patients | 1 | 2 | 3 | 4 | 5 |
| I think this rotation will be important for my residency training and education | 1 | 2 | 3 | 4 | 5 |
| I feel confident in leading an inpatient medicine ward team | 1 | 2 | 3 | 4 | 5 |

3. Please rate the amount of prior teaching you have received on the following topics

|  | None | Too Little | About Right | Too Much | Far Too Much |
| --- | --- | --- | --- | --- | --- |
| Patient Safety | 1 | 2 | 3 | 4 | 5 |
| Quality Improvement | 1 | 2 | 3 | 4 | 5 |
| Hospital Metrics | 1 | 2 | 3 | 4 | 5 |
| Hospital Reimbursement | 1 | 2 | 3 | 4 | 5 |
| Cost-Conscious Care | 1 | 2 | 3 | 4 | 5 |
| Physician Billing and Coding | 1 | 2 | 3 | 4 | 5 |
| Discharge Planning/Transitions of Care | 1 | 2 | 3 | 4 | 5 |

4. Please rate your knowledge about the following topics

|  | Very Poor | Poor | Fair | Good | Excellent |
| --- | --- | --- | --- | --- | --- |
| Patient Safety | 1 | 2 | 3 | 4 | 5 |
| Quality Improvement | 1 | 2 | 3 | 4 | 5 |
| Hospital Metrics | 1 | 2 | 3 | 4 | 5 |
| Hospital Reimbursement | 1 | 2 | 3 | 4 | 5 |
| Cost-Conscious Care | 1 | 2 | 3 | 4 | 5 |
| Physician Billing and Coding | 1 | 2 | 3 | 4 | 5 |
| Discharge Planning/Transitions of Care | 1 | 2 | 3 | 4 | 5 |

5. Please rate your level of agreement with the following statements

|  | Strongly Disagree | Disagree | Neither Agree Nor Disagree | Agree | Strongly Agree |
| --- | --- | --- | --- | --- | --- |
| I understand what a hospitalist does | 1 | 2 | 3 | 4 | 5 |
| I am interested in becoming a hospitalist or applying for a job with combination hospitalist work | 1 | 2 | 3 | 4 | 5 |
| I think hospitalists are an integral part of inpatient medicine hospital care | 1 | 2 | 3 | 4 | 5 |
