# Supplementary material for: Implementation of a Hospital Medicine Rotation and Curriculum for Internal Medicine Residents
Source: MedEdPORTAL. 2020 Sep 29;16:10977. doi: 10.15766/mep_2374-8265.10977 (PMC7526505; doi:10.15766/mep_2374-8265.10977)
Supplement: Supplementary file 1 — RITE Orientation Email.docxPre-RITE Survey.docxPost-RITE Survey.docxModule 1 Patient Safety.docxModule 2 QI, Metrics, Reimbursement, & Care.docxModule 3 Physician Billing & Coding.docxModule 4 Transitions of Care.docx [file mep_2374-8265.10977-s001.zip › C. Post-RITE Survey.docx]

**Post-RITE Survey**

1. Please choose you level of agreement with each of the following statements

|  | Strongly Disagree | Disagree | Neither Agree Nor Disagree | Agree | Strongly Agree |
| --- | --- | --- | --- | --- | --- |
| I feel confident independently managing hospitalized medicine patients | 1 | 2 | 3 | 4 | 5 |
| I think this rotation was important for my residency training and education | 1 | 2 | 3 | 4 | 5 |
| I feel confident in leading an inpatient medicine ward team | 1 | 2 | 3 | 4 | 5 |
| I think this rotation will help me when I am the upper level on a ward team | 1 | 2 | 3 | 4 | 5 |

2. Please rate the teaching you received on the following topics while on the RITE team

|  | None | Too Little | About Right | Too Much | Far Too Much |
| --- | --- | --- | --- | --- | --- |
| Patient Safety | 1 | 2 | 3 | 4 | 5 |
| Quality Improvement | 1 | 2 | 3 | 4 | 5 |
| Hospital Metrics | 1 | 2 | 3 | 4 | 5 |
| Hospital Reimbursement | 1 | 2 | 3 | 4 | 5 |
| Cost-Conscious Care | 1 | 2 | 3 | 4 | 5 |
| Physician Billing and Coding | 1 | 2 | 3 | 4 | 5 |
| Discharge Planning/Transitions of Care | 1 | 2 | 3 | 4 | 5 |

3. Please rate your knowledge about the following topics

|  | Very Poor | Poor | Fair | Good | Excellent |
| --- | --- | --- | --- | --- | --- |
| Patient Safety | 1 | 2 | 3 | 4 | 5 |
| Quality Improvement | 1 | 2 | 3 | 4 | 5 |
| Hospital Metrics | 1 | 2 | 3 | 4 | 5 |
| Hospital Reimbursement | 1 | 2 | 3 | 4 | 5 |
| Cost-Conscious Care | 1 | 2 | 3 | 4 | 5 |
| Physician Billing and Coding | 1 | 2 | 3 | 4 | 5 |
| Discharge Planning/Transitions of Care | 1 | 2 | 3 | 4 | 5 |

4. Please rate your level of agreement with the following statements

|  | Strongly Disagree | Disagree | Neither Agree Nor Disagree | Agree | Strongly Agree |
| --- | --- | --- | --- | --- | --- |
| I understand what a hospitalist does | 1 | 2 | 3 | 4 | 5 |
| I am interested in becoming a hospitalist or applying for a job with combination hospitalist work | 1 | 2 | 3 | 4 | 5 |
| I think hospitalists are an integral part of inpatient medicine hospital care | 1 | 2 | 3 | 4 | 5 |

5. Compared to when you started the rotation, how has your interest in hospital medicine changed?

- Significantly less interested
- Slightly less interested
- Same level of interest
- Slightly more interested
- Significantly more interested

6. Please rate the following in influencing your level of interest in hospital medicine

(1= LEAST influential/ 5= MOST influential)

| Salary | 1 | 2 | 3 | 4 | 5 |
| --- | --- | --- | --- | --- | --- |
| Job Satisfaction | 1 | 2 | 3 | 4 | 5 |
| Work/Life Balance | 1 | 2 | 3 | 4 | 5 |
| Patient Diversity | 1 | 2 | 3 | 4 | 5 |
| Pursuing a Fellowship | 1 | 2 | 3 | 4 | 5 |
| Ease of finding a job and/or relocating | 1 | 2 | 3 | 4 | 5 |
| Pursuing a Primary Care outpatient position | 1 | 2 | 3 | 4 | 5 |

Other MOST influential not listed above:

7. Please describe what you LIKED about the rotation (at least one item)

8. Please describe what you DID NOT LIKE about the rotation (at least one item)

9. The RITE curriculum was presented in the workbook that you read independently. Each week, the attending should have led a group discussion about how to apply the information you learned. What method do you prefer for the curriculum before the group discussion?

- Just the way it is—workbook
- Online videos
- Lecture/didactics
- Powerpoint slides
- Reading primary journal articles
- Other (please specify)

10. Any final comments, suggestions, or feedback about the RITE rotation and curriculum?
