# Supplementary material for: Implementation of a Hospital Medicine Rotation and Curriculum for Internal Medicine Residents
Source: MedEdPORTAL. 2020 Sep 29;16:10977. doi: 10.15766/mep_2374-8265.10977 (PMC7526505; doi:10.15766/mep_2374-8265.10977)
Supplement: Supplementary file 1 — RITE Orientation Email.docxPre-RITE Survey.docxPost-RITE Survey.docxModule 1 Patient Safety.docxModule 2 QI, Metrics, Reimbursement, & Care.docxModule 3 Physician Billing & Coding.docxModule 4 Transitions of Care.docx [file mep_2374-8265.10977-s001.zip › D. Module 1 Patient Safety.docx]

Module 1: Medicare Basics

Module 1: Medicare Basics

Module 1: Medicare Basics

Module 1: Medicare Basics

Module 1: Basic Principles in Patient Safety

Module 1: Medicare Basics

Module 1: Medicare Basics

*Learning Objectives*

- Explain the extent of medical errors and the related costs in hospital medicine
- Define medical errors and describe how errors reach the patient
- Categorize medical errors as sentinel events, near misses, or other adverse events
- Categorize medical errors as diagnostic, therapeutic, preventive, or other
- Provide examples of the types of errors in hospital practice

**Module 1: Basic Principles in Patient Safety**

**Pre-lecture Assignment:**

- Review the clinical scenario below and reflect on a few patient safety issues that are addressed. Also, develop a list of current patients you are managing who may have encountered safety issues during their hospitalization.
- Any websites provided in the module are optional reading

**Clinical Scenario: Ms. Smith and Safety**

After graduating from Internal Medicine residency, you decide to join a Hospital Medicine group in AwesomeTown, Texas. The hospital administration asks that you join the Patient Safety Committee. Since you are the “new” person, it would be rude to say no. To prepare for the upcoming meeting, you begin to read the first case:

*Ms. Smith is a 70-year-old female with a history of dementia who is admitted from a nursing home for erythema and warmth of her right lower extremity. She has a mild leukocytosis. The emergency room physician starts vancomycin for cellulitis and places a Foley catheter because she cannot ambulate to the restroom. The hospitalist admits the patient and places her on fall precautions. Two days into the hospitalization, her bed rail is accidentally left down after physical therapy. She has an episode of confusion and disorientation that evening and tries to get herself out of bed and falls to the floor. Luckily, the nurse finds her on the floor awake and able to answer questions. After another couple of days of intravenous antibiotics, her cellulitis seems to be clinically improving, but she begins having fevers and her leukocytosis is increasing again. The hospitalist sends for a urinalysis (UA), urine culture, blood culture and chest x-ray (CXR). Her blood culture and CXR are negative but her UA is now positive for pyuria and her urine culture grows Escherichia coli sensitive to ceftriaxone. She is started on antibiotics. After further review of the chart, you notice that she has had a Foley catheter in since admission. The catheter is not removed until hospital day 5. On hospital day 7, she is clinically improving and is discharged back to her nursing home to complete the antibiotic course.*

Since you are the only hospitalist physician on the Patient Safety Committee, the other members ask for your input as to how these adverse events could have been prevented.

## How Safe (or Hazardous) is our Health Care?

The case above provides an example of a medical error that led to significant harm. Is this an isolated event or a systematic problem? How safe is our health care?

Physicians often abide by the central principle “first, do no harm,” but are we really following this philosophy?

As a Harvard professor and a patient safety advocate and expert, Dr. Lucian Leape, compared mortality rates among various high-risk industries. He found that health care errors fell in the dangerous category alongside mountain-climbing while flying on scheduled airlines or riding on European railroads was ultra-safe.^1^

The estimate for deaths caused by medical errors varies widely depending on the source. Based on the 1999 Institute of Medicine (IOM) report, the estimate is between 44,000 to 98,000 deaths EACH YEAR! The number of deaths is equivalent to one jumbo jet crashing every day. Even more alarming – most of these deaths can be prevented.^2^ In 2013 “[A New, Evidence-Based Estimate of Patient Harms Associated with Hospital Care](https://journals.lww.com/journalpatientsafety/Fulltext/2013/09000/A_New,_Evidence_based_Estimate_of_Patient_Harms.2.aspx)” estimated more than 400,000 deaths due to medical error per year.^3^ More recent data from Johns Hopkins’ patient safety experts have calculated that more than 250,000 deaths per year are due to medical errors making it the third leading cause of death in the United States.^4^ Even with the wide variation in numbers, hospital administrators have understood the importance of medical errors and implemented many initiatives to improve patient safety. Think about how much money, effort, research and residency training are spent on these initiatives. Hopefully, after completing this module you will have a better perspective on how to reduce medical errors.

But I’m just a resident – what can I do about this? Stay tuned…this module will teach you the skills to make a significant impact on patient care. In addition, hospital administrators are eagerly seeking leaders in patient safety when hiring physicians. Some institutions like ours have even developed dedicated post-graduate training in quality improvement and patient safety. We will also discuss why hospitals are so interested in patient safety and quality improvement – sure, administrators care about the patients, but this also impacts the hospital’s revenue. Yes…this is important – for patient care, for reimbursements, and potentially for your future career.

## The Institute of Medicine Investigates

In 2000, the IOM investigated patient safety issues in the health care delivery system. They issued a groundbreaking report entitled *To Err is Human: Building a Safer Health System*.^2^ Let us discuss a few of the topics that were addressed in the report:

How significant are the costs associated with medical errors in the hospital?

Are errors caused by individual recklessness?

How can you categorize medical errors?

What are four recommended strategies for improvement?

## How do errors reach the patient?

As the title of the report suggests, humans are not infallible – we all make mistakes. We have to build systems to catch these mistakes before patients are adversely affected. The health care system represents one of the most complex systems in the world. What is the definition of a complex system?

The British psychologist James T. Reason first described the “Swiss Cheese” model. Each slice of cheese represents an individual part to a system and the holes represent weaknesses that continually change in size and orientation. When all the “holes” (weaknesses) align perfectly, it creates “a trajectory of accident opportunity.”^5^

Author Created Image

Let’s take the real-life story of Betsy Lehman as an example. Betsy Lehman was a well-known journalist for the Boston Globe since 1982 and authored a section on new developments in medicine entitled “Health Sense”.^6^ She was diagnosed with breast cancer in 1993 and was receiving care at the world-renowned Dana Farber Cancer Institute. Participating in a clinical trial, Betsy was receiving dose escalating treatment with cyclophosphamide.

Per the trial parameters, she should have received 1,000mg/m^2^ body surface area for four days. Instead, the Oncology fellow wrote “**4,000mg/m^2^ x 4 days**”. There was no attending oversight for the order. Betsy received four times the correct dose and died related to complications. Over 30 health care workers including physicians, nurses, and pharmacists were involved in her care. The mistake was only discovered when the data was being entered into a database for the clinical trial. Can you see all the “holes” in the Swiss Cheese for this case?

[Same picture with barriers listed]

Essentially, there was no double check or error check by the other components of this complex system – attending, nurses, pharmacists, and study personnel. Even her clinical signs of cyclophosphamide toxicity did not draw anyone’s attention. The other big problem is that the error was not even detected until 3 months later. How many other patients have been similar victims? If this can happen to a well-educated woman like Betsy Lehman at a world-renowned hospital like Dana Farber, then this can happen anywhere.

Okay…hopefully now you understand that medical errors are a bigger problem than you first thought. Let me guess what your next question is…

##

## What is the role of the hospitalist in patient safety?

Hospitalists are at the front lines with health care delivery within the complex hospital environment. A well-trained hospitalist understands the complex system including all the vulnerabilities (the “holes”). Medical administrators are increasingly asking hospitalists to serve on important quality and patient safety committees to detect weaknesses and implement strategies to prevent patient harm. Think about all the problems and workarounds that you know about when working in your hospital.

Would Betsy Lehman receive the overdose of cyclophosphamide in today’s hospital system? Hospital leaders working in multidisciplinary teams have developed safeguards to prevent similar medical errors. At most institutions, all chemotherapy orders are reviewed and cosigned by an attending physician, all calculations are checked by the pharmacist, and some electronic medical record (EMR) systems generate red flag alerts if medical dosing exceeds the normal ranges.

## Let’s Practice: “Swiss Cheese” model for Ms. Smith

Thinking back to Ms. Smith’s case, describe at least three “holes” in the Swiss Cheese model.

1. _______________________________________________________________________
2. _______________________________________________________________________
3. _______________________________________________________________________
4. _______________________________________________________________________
5. _______________________________________________________________________

##

## Understanding medical errors

What exactly are medical errors? Different organizations have slightly different definitions. The IOM report defines medical errors as “the failure of a planned action to be completed as intended or the use of a wrong plan to achieve an aim.”^2^

Medical errors can be categorized in several different ways. Let’s look at one approach here. Medical errors can be subdivided into near misses and adverse events. A **near miss** represents an event or situation that did not produce patient injury, but only because of chance. This may be related to a patient factor or being “caught” by the system. In other words, something bad *could have* happened. For example, a patient with Chronic Kidney Disease (CKD) Stage V was prescribed full dose enoxaparin and the pharmacists or EMR system provided an alert that prevented the administration of enoxaparin. Dr. Leape defines **adverse events** as “an unintended injury that was caused by medical management and that resulted in measurable disability.”^7^ In other words, something bad happened to the patient. For example, a patient with CKD Stage V was given full dose enoxaparin, and then had significant bleeding during surgery.

| Examples of Adverse Events | |
| --- | --- |
| - Wrong site surgeries | - Restraint-related injuries or death |
| - Patient falls | - Mistaken patient identities |
| - Patient elopement | - Surgical injuries |
| - Improper blood transfusions | - Adverse drug events |

**Sentinel events** are a subcategory of adverse events that lead to death or serious physical or psychological injury. Many people call these the “never” events – as a hospital system, we should never let this happen again. All sentinel events are preventable. For example, a patient with CKD Stage V was prescribed subcutaneous heparin for prophylaxis. Instead, the patient was given subcutaneous potassium chloride resulting in skin necrosis requiring skin grafts.

Unpreventable

Preventable

Medical Errors

##

## Author created image

## Can all adverse events be prevented?

The Leape article looked into a random sampling of over 30,000 hospital records to discover 1133 patients representing 3.7% with disabling injuries related to medical treatment.^7^ The article gave examples of medical errors that were neither preventable nor predictable including idiosyncratic drug reactions or postoperative myocardial infarctions in young patients without previous evidence of heart disease. It also discusses other unpreventable adverse events that occurred with predictable frequency, however patients accepted the risk because of the potential benefits such as chemotherapy. He goes on to say that the majority of adverse events are preventable particularly those due to human error which are common in medical practice.^7^

Hospitals continue to implement patient safety interventions. Here are a few examples that are probably familiar to you:

| Patient Safety Interventions |
| --- |
| - After visit summaries to give patients an updated medication list |
| - Red alerts when prescribing medications to patients with documented allergies |
| - Creatinine and pregnancy test (when appropriate) checked by a radiology technician before computed tomography scans |
| - Attending co-signature for high-risk medication administration (ex: chemotherapy) |
| - “Time Out” protocol prior to any invasive procedure |

Dr. Leape provides another way to characterize errors by using 4 categories: diagnostic, treatment, preventive, and other.^8^

**1) Diagnostic**

Error or delay in diagnosis

Failure to employ indicated tests

Use of outmoded tests or therapy

Failure to act on results of monitoring or testing

**2) Treatment**

Error in the performance of an operation, procedure, or test

Error in administering the treatment

Error in the dose or method of using a drug

Avoidable delay in treatment or in responding to an abnormal test

Inappropriate (not indicated) care

**3) Preventive**

Failure to provide prophylactic treatment

Inadequate monitoring or follow-up of treatment

**4) Other**

Failure of communication

Equipment failure

Other system failure

## Let’s Practice: Categorize these errors

|  | Diagnostic | Treatment | Preventive | Other |
| --- | --- | --- | --- | --- |
| 1) The resident checks out his patients to the overnight float and fails to ask the float to check the BMP at 10pm. The patient has worsening hyperkalemia and goes into ventricular tachycardia at 2am. |  |  |  |  |
| 2) The resident starts vancomycin at 1gram q12 hours for cellulitis. The patient develops acute renal failure with creatinine trending up to 4.0. A vancomycin level drawn five days into the patient’s stay is >100. |  |  |  |  |
| 3) A 65 y/o patient visits the resident in outpatient clinic. She has newly diagnosed iron deficiency anemia and the patient notes a change in the caliber of her stools. She is sent home with only iron supplementation. No referral is made. |  |  |  |  |
| 4) Within 24 hours, 35 patients are admitted in need of emergent hemodialysis. Due to the limitation of dialysis machines, only 20 patients underwent dialysis. One of the patients who had to wait develops worsening volume overload and needs to be intubated. |  |  |  |  |
| 5) A 34 y/o man undergoes a splenectomy after abdominal trauma. He is not given any vaccinations and returns in 3 months with pneumococcal sepsis. |  |  |  |  |
| 6) A 55 y/o man is admitted for shortness of breath, hypoxia, and tachycardia. Antibiotics are started for treatment of pneumonia despite no infiltrates on CXR. Three days later, a CT Chest reveals a large pulmonary embolus. |  |  |  |  |
| 7) A 67 y/o woman is admitted for a symptomatic left pleural effusion. The resident attempts a thoracentesis of the right pleural space resulting in a pneumothorax. |  |  |  |  |

## Answers: 1) Treatment and Other- Failure of Communication 2) Treatment and Preventive 3) Diagnostic 4) Treatment and Other- System Failure 5) Preventive 6) Diagnostic 7) Treatment

## Creating a Culture of Patient Safety

Systems can only be improved if medical errors can be discussed openly without fear of reprisal. The hospital should promote a culture of patient safety. As explained above, human error will happen and systems need to be in place to prevent that error from affecting the patient. With that philosophy, when errors do occur that affect the patient, it is not an *individual’s* fault, but rather a failure of the systems.

Let me give you an example from the airline industry. A baggage handler working for Safety First Airways was loading up the luggage into the cargo area of the airplane. He finished closing all the doors and the plane began to move to the runway for takeoff. That’s when he saw something that just did not look right near one of the wings. He had loaded thousands of aircrafts and he thought there was something wrong. The pilot was notified and the takeoff was aborted. The plane returned to the jet-way and a thorough inspection revealed no abnormalities. What action was taken on the baggage handler? The company gave him an award for reporting his suspicion!

What would happen with a similar situation in medicine?

## Facilitated Exercise

Your facilitator will help guide you through the following exercises.

Please return to the initial clinical scenario with Ms. Smith and discuss the following questions.

1) How would you categorize the medical error(s)?

2) Brainstorm the possible causes (both individual and systems) that may have led to this error.

3) For the Patient Safety Committee, please outline your suggestions for change to prevent this type of error from happening again.

4) How should the committee discuss the adverse event with the discharging hospitalist? Should there be any adverse action taken against the physician?

5) Provide 2-3 examples of patient safety related errors that you have seen during your residency training?

**Teaching Points for the Questions:**

1. As often happens, there were many errors with care. There was a treatment and diagnostic error with placing a foley that was not indicated. There was a failure of communication or preventive error when the bed rail was left down. There was a preventive error when the foley was left in place for many days.
2. Many factors can lead to these errors – including knowledge of appropriate indications for foley placement, communication issues between therapist and nurse, and lack of policy or hospital guidelines around discontinuation of unnecessary catheters.
3. It is important to emphasize not just education of staff, but stronger interventions, such as early discontinuation of lines/foleys that could be implemented on a systems level.
4. Feedback to all the staff involved is important so that people can learn and contribute to solutions. Since many systems issues led to this event, it would not be appropriate to take adverse action against the physician.
5. If no errors come to mind, encourage residents to think about issues they may have encountered around communication, over testing, or over treating.

## Conclusion

Almost twenty years after the IOM report *To Err is Human*, improving patient safety remains a top priority in our health care system. We have to remember that keeping our hospitals safe requires teamwork and a multidisciplinary approach if we want to see significant change. Hospitalists and residents are now at the forefront of leading initiatives to improve patient safety and quality of care in hospitals.

**References**

1. Lucian Leape, MD, presentation at 2012 Symposium on Human Factors and Ergonomics in Health Care. Baltimore, MD.
2. Kohn LT, Corrigan JM, Donaldson MS. *Institute of Medicine 2000. To Err Is Human: Building a Safer Health System*. Washington, DC: The National Academies Press.
3. James JT. A new, evidence-based medicine estimate of patient harms associated with hospital care*. J of Patient Saf.* 2013;9(3):122-128.
4. Makary MA, Daniel M. Medical error the third leading cause of death in the US. *BMJ.* 2016;353:i2139.
5. Reason J. Human error: models and management. *West J Med.* 2000; 172(6):393-6.
6. Bohmer RM, Winslow A. ["Dana-Farber Cancer Institute, The."](http://hbr.org/product/Dana-Farber-Cancer-Instit/an/699025-PDF-ENG) Harvard Business School Case 699-025, March 1999. [Revised July 1999].
7. Leape LL, Brennan TA, Laird N, et al. The nature of adverse events in hospitalized patients. results of the harvard medical practice study II. *N Engl J Med*. 1991;324(6):377-384.
8. Leape LL, Lawthers AG, Brennan TA, Johnson WG. Preventing medical injury. QRB Qual Rev Bull. 1993;19(5):144-149.
