# Supplementary material for: Implementation of a Hospital Medicine Rotation and Curriculum for Internal Medicine Residents
Source: MedEdPORTAL. 2020 Sep 29;16:10977. doi: 10.15766/mep_2374-8265.10977 (PMC7526505; doi:10.15766/mep_2374-8265.10977)
Supplement: Supplementary file 1 — RITE Orientation Email.docxPre-RITE Survey.docxPost-RITE Survey.docxModule 1 Patient Safety.docxModule 2 QI, Metrics, Reimbursement, & Care.docxModule 3 Physician Billing & Coding.docxModule 4 Transitions of Care.docx [file mep_2374-8265.10977-s001.zip › E. Module 2 QI, Metrics, Reimbursement, & Care.docx]

Module 2: Quality Improvement, Hospital Metrics/Reimbursement, and Cost-Conscious Care

*Learning Objectives*

- Define quality in medical care according to the AHRQ
- List the IOM six aims of quality health care
- Design a quality improvement project using the PDSA cycle
- Differentiate observation and inpatient status
- Describe how hospitals are reimbursed
- Explain how physician documentation affects hospital reimbursement and performance metrics
- Describe quality based adjustments to hospital reimbursement
- Summarize how hospital metrics are shared with the general public
- Apply cost-conscious care to daily practice

**Module 2: Quality Improvement, Hospital Metrics/Reimbursement, and Cost- Conscious Care**

- Any websites provided in the module are optional reading

**Introduction**

How do you define quality health care? The US Agency for Healthcare Research and Quality (AHRQ) describes quality health care as “doing the right thing, at the right time, in the right way, for the right person—and having the best possible results.” As hospitalists, we often see the broken parts in the system. Can we do better? YES! Possessing quality improvement (QI) skills will help make this possible. The Institute of Medicine (IOM) provides six aims for QI which we will discuss in this module. We will also take a closer look at the Institute for Healthcare Improvement (IHI) Model which includes the Plan-Do-Study-Act (PDSA) cycle. As the quality movement has spread through medicine, we will investigate the common performance and quality metrics currently being measured by hospitals. Health care delivery requires time, money, and other resources. We will explore whether we can provide equal or better quality of health care while decreasing overall costs to the system. In other words, can we eliminate the waste in medical care?

We have already discussed problems with patient safety in the first module and using QI methods, hospitals and physicians are working together to change the systems.

Here is another way to think about QI. Over time, our scientific knowledge has grown at a very rapid pace. The consistent implementation of scientific evidence into actual practice is still lacking. QI aims to bridge that gap to improve the effectiveness and efficiency of health care.

**Why is QI Important?**

According to the AAP EQUIPP QI basics module, QI uses a formal systematic approach to continuously improve patient care in a measurable way and outcomes depend on the quality of care received.

**IOM Six Aims for Quality Improvement**

Shortly after releasing their report on patient safety, the IOM addressed a strategy to improve health care delivery in the US by developing the Six Aims.^1^ You can remember the Aims with the pneumonic “STEEEP” as illustrated in the diagram.

- What are the Six Aims defined by the IOM?

| **S**afe | Avoiding harm from care |
| --- | --- |
| **T**imely | Avoiding delays for those who receive and give care |
| **E**ffective | Avoiding overuse and misuse of services |
| **E**fficient | Avoiding waste including waste of energy, ideas, equipment, supplies |
| **E**quitable | Providing care that does not vary because of personal characteristics |
| **P**atient-Centered | Providing care that is respectful of and responsive to patient preferences, needs and values |

These aims provide the most influential framework for quality assessment and have guided measure development initiatives in the health care sector.

## Models for Quality Improvement

Many of the models for quality improvement have been borrowed from other industries. The Varkey article “Basics of Quality Improvement in Health Care” provides a concise overview of the fundamentals of QI methods and terminology.^2^  Key methods discussed in the article are the PDSA, six-sigma, and lean strategies. Here we will focus on the PDSA cycle as it is the most commonly used method for rapid improvement in health care.

What are the four steps of the PDSA cycle?

1. _____________________________________________________
2. _____________________________________________________
3. _____________________________________________________
4. _____________________________________________________

Author created image

**Clinical Scenario: PDSA and Pulmonary Embolism (PE) studies**

Hospital leadership has come to you as the quality leader in your hospitalist group to help develop a method to decrease unnecessary ordering of CT chest PE studies if the clinical suspicion, pre-test probability, and D-dimer is low or not ordered. The National Quality Forum (NQF) has endorsed a performance measure to increase imaging efficiency of these studies in the emergency department.

The scenario suggests that avoidable imaging studies are being done in patients with a low pretest probability of PE and a D-dimer that was negative or not ordered.

Let’s walk through how you would design a QI project to reduce inappropriate CT PE imaging for low-risk patients, using the PDSA model.

**PLAN**

1. What are we measuring? What outcomes do we want to improve?
2. Collect baseline data about this outcome at your own hospital.
3. What system changes do you propose to improve this outcome?
4. Which different departments should be involved with this system change?
5. Estimate the effect of the system change on the outcome being measured.
6. Are there any “side effects” to this system change?

**DO**

1. Implement the plan that you have developed
2. Continue to measure the outcome

**STUDY**

1. Analyze the data and summarize the effect

**ACT**

1. Based on the pilot project, outline other changes that need to be made
2. Go back to the PLAN and restart the cycle.

**Example Answers to the Clinical Scenario**

**PLAN**

1. What are we measuring? What outcomes do we want to improve?
*Percentage of CT PE protocol scans done on patients with low pretest probability that have a negative D-dimer or D-dimer not ordered*

2. Collect baseline data about this outcome at your own hospital.
*Hospital Wasteful provided you with all their current data. Over one month, they did 1000 CT PE protocol scans. Of these patients, 100 patients were classified as low pretest probability of PE. Out of these 100 patients -- 20 patients had no D-dimer and 60 patients had a negative D-dimer. So…~80% of CT PE protocol scans on low risk probability patients was inappropriate.*

3. What system changes do you propose to improve this outcome?
*When a physician enters an order for a CT PE protocol into the EMR system, a checklist will be provided for the physician to fill out a probability classification and ask them to order a D-dimer.*

4. From the hospital, which different departments should be involved with this system change?
*Radiology, ER physicians, IM physicians, IT support, Lab representatives, Nursing*

5. Estimate the effect of the system change on the outcome being measured.
*We hope to reduce inappropriate CT PE protocol scans from 80% to 20%*

6. Are there any “side effects” to this system change?
*If physicians think a D-dimer has to be ordered on every patient before getting a CT PE study, it may lead to excessive ordering of D-dimers when it may not be necessary.*

**DO**

1. Implement the plan that you have developed
2. Continue to measure the outcome

**STUDY**

1. Analyze the data and summarize the effect
   *After implementation at Hospital Wasteful, the rate of inappropriate CT PE protocol scans went from 80% to 50%. This did not achieve the drop that was estimated. You interviewed everyone involved to better understand what was happening.

   Based on interviews with the physicians, they found the checklist and scoring system too cumbersome and interfered with their workflow. Many times, they would just click the boxes without reading them to get to the next screen. Also, they wanted an order that said “Check D-dimer. If positive, then CT PE protocol”. This would streamline their workflow.*

**ACT**

1. Based on the pilot project, outline other changes that need to be made
   *Feedback was very important in this situation to understand the problems. The team met again to discuss further changes that are needed to improve the outcome.*
2. Go back to the PLAN and restart the cycle with your next action.

## What is your QI Project?

The scenario above provides an excellent example of a project that helps reduce cost, improve patient safety and provide better patient care. During this module, think about some measurable outcomes that need to be improved based on your experience in the hospital. Think about the Six Aims outlined by IOM and the PDSA method to outline your project.

**Hospital Metrics and Reimbursement**

**Clinical scenario: Trouble with Utilization Management**

You are a newly hired hospitalist and excited to start your first week. After completing a grueling three-year residency and passing the boards you are confident in your skills. It is not even 9AM and your assigned case manager is paging you.

Case Manager: “Doc, I see that you placed Mr. Sunshine in acute but he does not meet criteria. Can you justify this? The insurance company is going to deny payment and Mr. CEO is not going to be happy!”

## Level of Care: Observation versus Inpatient Status

Per CMS, “observation care is a well-defined set of specific, clinically appropriate services, which include ongoing short term treatment, assessment, and reassessment before a decision can be made regarding whether patients will require further treatment as hospital inpatients or if they are able to be discharged from the hospital.”^3^

Although physically in the hospital, patients in observation status are considered outpatients. Labs, diagnostic test, and physician services are billed as if the patient was being seen in clinic. In general, the total payment to hospitals is lower for observation stays in comparison to acute. If a patient placed in acute status does not have medical necessity (meet criteria) insurers can deny payment. They do not reduce the payment to the appropriate level of care, but instead, the hospital receives no compensation for the care provided.

**How do you choose between observation and acute?**

Physicians should use clinical judgment to risk stratify patients based on risk of adverse outcomes and expected duration of stay. Per the two midnight rule, if a patient is expected to be in the hospital less than two midnights they should be in observation status.

Hospital utilization reviewers and insurers use commercial products to objectively determine the appropriate level of service based on the patients presenting problem. Guidance from your utilization review/case management nurses can help to select the appropriate level of care.

Some disagreement between physicians and these commercial criterions is expected. It is vital to justify and document your reasoning with clinical evidence in case of payment denial.

**CASE BASED PRACTICE**

For the following case examples decide if you believe that the patient should be placed in observation or acute and justify your decision.

1. 55 y/o M with PMH of chronic lower back pain, previous cocaine use, and hyperlipidemia who presents with left-sided non-exertional chest pain.

Temp 98.5, HR 99, RR 12, BP 170/85, O_2_ sat 100% on RA

EKG: normal sinus rhythm, no ST or T-wave changes

Trop: 0.02

1. 53 y/o M with PMH of CHF (EF 35-39%) who presents from clinic with pedal edema bilaterally and dyspnea with walking up a flight of stairs. Also with orthopnea and PND.

Temp 97.8, HR 85, RR 12, BP 140/67, O_2_ sat 94% on RA

Exam: minimal bibasilar crackles, 2+ pitting edema of BLE.

1. 66 y/o M with PMH of CAD, DM who presents with cellulitis of the RLE for one week. Has been on oral antibiotics for 2 days and redness continues to spread.

Temp 98.1, HR 66, RR 12, BP 122/60, O_2_ sat 99% on RA

Exam: Erythema of RLE from mid-shin to top of ankle, increased warmth

1. 70 y/o M who presents to the ER with cough and shortness of breath for the past one week.

Temp 99.4, HR 85, RR 16, BP 122/60, O_2_ saturation 92%

CXR with RLL pneumonia.

1. 60 y/o F with COPD presents with worsening SOB and productive cough for the past 3 days. She has received three nebulizer treatments and prednisone in ED with minimal improvement.

Temp 98, HR 82, BP 123/84, RR 16, O_2_ sat 89% on RA

1. 66 y/o F presented with 2 day history of lightheadedness, mid-epigastric abdominal pain, and dark stool.

BP 110/60, HR 90, positive orthostatic vitals

Hgb 7.8, BUN 18, Cr 1.0

**ANSWERS**

Case management suggested level of care:

1. Observation - This patient is presenting with atypical or non-cardiac chest pain. All patients admitted for “chest pain rule out” should be placed in observation.

2. Observation - This patient is presenting with a heart failure exacerbation. The patient does not have hypoxemia or hemodynamic instability to warrant acute admission.

3. Acute – This patient is presenting with cellulitis which has failed appropriate outpatient therapy. The failure of oral antibiotics and need for IV antibiotics warrants inpatient admission.

4. Observation - This patient is presenting with pneumonia without hypoxemia or hemodynamic instability to warrant acute admission.

5. Acute – This patient is presenting with a COPD exacerbation. The patient has significant tachypnea and hypoxemia which warrants inpatient admission.

6. Acute – This patient is presenting with an acute GI bleed. The patient has signs of hemodynamic instability and positive orthostatic vital signs which warrants inpatient admission.

**INPATIENT PROSPECTIVE PAYMENT SYSTEM**

Private insurers pay hospitals based on pre-negotiated rates with hospitals. These rates are adjusted for severity of illness and hospital day. This system lacks uniformity and reimbursement rates are variable between various hospitals and insurers.

In this discussion we will focus on how hospitals are paid by Medicare, which is known as the Inpatient Prospective Payment System (IPPS). In this system, hospitals receive a single payment for each individual hospitalization. Medicare categorizes each hospitalization into a diagnosis related group (DRG). The primary DRG determinants are the principal diagnosis, procedures, and patient demographics (Figure 1). This is further subdivided by the presence and severity of complications and co-morbidities into medical severity diagnosis related groups (MS-DRG). These conditions are divided into three groups: major complication/co-morbidity (MCC), complication/co-morbidity (CC), or no CC/MCC based on yearly determinations by CMS (Figure 2). As of 2017, there are 757 specific MS-DRG’s that an inpatient stay can be classified to.^4^

ICD-10 Principal Diagnosis

ICD-10 Procedure Codes

+

DRG

=

+

Patient Demographics

+

or

***Figure 1: Components of Diagnosis Related Group Classification***

MCC

CC

No CC or MCC

DRG

or

MS- DRG

or

+

=

***Figure 2: Components of Medical Severity Diagnosis Related Group Classification***

Associated with each DRG, there are several values generated based on historical data from CMS.

- Relative Weight – A numerical value that reflects the relative costliness of care
- Length of Stay (LOS) – Number of days a patient in each DRG is expected to be hospitalized. There are two different LOS calculations: average LOS and geometric LOS, which is modified to dampen the effects of outliers.
- Reimbursement – The amount a hospital will be reimbursed for the inpatient care. This varies between hospitals and hospital systems based on many factors including: geographic location, presence of resident trainees, and proportion of uninsured and low-income patients.

In addition to the DRG assignment, principal and secondary diagnoses are used to calculate individual patient’s severity of illness and risk of mortality. These are the basis of the hospital’s risk adjusted mortality ratio.

Coders can only code what you document. If you do not document thoroughly and accurately, the coders are limited in the ICD-10 diagnoses that they can select. Inaccurate physician documentation results in lower level MS-DRG assignment, lower relative weight, increased observed: expected length of stay, lower reimbursement, and falsely elevated mortality ratios.

**CASE EXAMPLE:**

Mr. Blue is a 68 y/o M with GOLD Stage 3 COPD who presented with 1 day of wheezing and shortness of breath and increased sputum production. In the ER, vital signs showed T 99°F, HR 112, BP 130/80, RR 25, O2 97% on 3L. He was in moderate respiratory distress using accessory muscles and pursed lips to breathe. ABG showed a pH7.26, pCO2 59, pO2 90 on 2L NC. He was placed on NIPPV and admitted to the MICU. He was started on steroids, nebulizers, and azithromycin. He improved over the next couple of days and was discharged home with optimization of COPD medication and to complete a course of steroids and antibiotics.

Discharge Diagnosis, as documented: COPD Exacerbation

Discharge Diagnosis, optimal: acute respiratory failure with hypercarpnia, COPD Exacerbation

Below are the MS-DRG and metrics based on actual versus optimized documentation:

|  | As Documented | Optimized |
| --- | --- | --- |
| MS-DRG | 192 | 189 |
| Relative weight | 0.72 | 1.22 |
| Average Length of Stay | 3.0 | 4.7 |
| Reimbursement | $3988 | $6695 |

Can you believe the staggering difference! Expected length of stay increased by 1.7 days and potential hospital reimbursement increased by more than $2,700. Imagine multiplying this by the number of admissions over a month or year. This has huge financial implications for the hospital. Most hospitals now have clinical documentation improvement teams to educate and assist physicians to optimize documentation. There are many opportunities to enhance documentation that you should learn prior to entering practice.

**Common Opportunities for Documentation Optimization**

Sepsis, Respiratory Failure, Heart Failure, Functional Quadriplegia, Acute Blood Loss Anemia, Malnutrition, Acute Tubular Necrosis, Altered Mental Status

**FACILITATED EXERCISE**

Look through a few of your progress notes together with your attending and review the documentation used in your primary and secondary diagnoses. Discuss improvements that can be made in the wording or placement of diagnoses in order to optimize documentation. You can take notes below:

_____________________________________________________________________________________

_____________________________________________________________________________________

_____________________________________________________________________________________

_____________________________________________________________________________________

_____________________________________________________________________________________

_____________________________________________________________________________________

**Clinical Scenario: Quality Costing Money**

You get a call from the chief quality officer. You are proud of yourself with the improved documentation and relative weights for your patients. You are sure that he is calling to thank you for all your hard work.

Chief Quality Officer: “Doc, we need to talk about your performance measures. Your patient satisfaction scores and patient safety indicators are in the dumps. What are we going to do about this? We are losing money and we will lose patients soon.”

**QUALITY BASED ADJUSTMENTS**

The IPPS is not the complete picture regarding hospital reimbursement for inpatient care. Starting in 2012, CMS added an additional modifier to facilities reimbursement rates based on the quality of care they provide.

There are three different programs that can lead to payment adjustments for hospitals:

1. Value based purchasing (VBP) program

Includes hospital quality data based on 21 measures in the domains of patient experience, safety, clinical care (process and outcomes), and efficiency and cost reduction (Table 1). Hospitals are assessed based on either their overall achievement or improvement from previous years. Facilities that exceed expectations are given monetary incentives that are deducted from lower performing facilities. CMS can penalize hospitals by up to a 2% reduction in DRG payments.^5^

| Measure description | domain |
| --- | --- |
| Catheter Associated UTI | Safety |
| Central Line-Associated Blood Stream Infection | Safety |
| Clostridium difficile infection | Safety |
| Methicillin-Resistant Staph Aureus Bacteremia | Safety |
| Surgical Site Infections: colon, hysterectomy | Safety |
| Acute MI 30-day mortality | Clinical Care - outcomes |
| Heart Failure 30-day mortality | Clinical Care - outcomes |

| Pneumonia 30-day mortality | Clinical Care - outcomes |
| --- | --- |
| Fibrinolytic therapy within 30 minutes | Clinical Care - processes |
| Influenza immunization | Clinical Care - processes |
| Elective Delivery Prior to 39 weeks | Clinical Care - processes |
| Medicare Spending per Beneficiary | Efficiency and Cost |
| HCAHPS Survey   1. Communication with nurses 2. Communication with doctors 3. Responsiveness of hospital staff 4. Pain management 5. Communication about medicines 6. Cleanliness and Quietness 7. Discharge information 8. Overall Hospital Rating | Patient and Caregiver-Centered Experience of Care/Coordination of Care |

Table 1: 2017 Value Based Purchasing Quality Metrics

1. Readmission reductions program

A program that evaluates facilities 30-day readmission rate for patients admitted with six specific diagnoses. Admissions to an acute care facility within 30 days from discharge are counted. Hospitals with excessive readmission can be penalized up to 3% of their DRG payment.^6^

| prinicipal diagnosis |
| --- |
| Acute Myocardial Infarction |
| Heart Failure |
| Chronic Obstructive Pulmonary Disease |
| Total Hip/Knee Arthroplasty |
| Coronary Artery Bypass Graft |
| Pneumonia |

Table 2: 2017 Readmission Reduction Principal Diagnoses

1. Healthcare Associated Complications (HAC) reduction program

A program that evaluates hospitals on their rates of hospital acquired infections and complications. See the table below for complications included in the measure. Hospitals in the bottom quartile of performance are penalized with a 1% reduction in their DRG payments.^7^

| Complication Description |
| --- |
| Pressure Ulcer |
| Iatrogenic Pneumothorax |
| In-Hospital Fall with Hip Fracture |
| Perioperative Hemorrhage or Hematoma |
| Postoperative Acute Kidney Injury Requiring Dialysis |
| Postoperative Respiratory Failure |
| Perioperative Pulmonary Embolism or Deep Venous Thrombosis |
| Postoperative Sepsis |
| Postoperative Wound Dehiscence |
| Unrecognized Abdominopelvic Accidental Puncture/Laceration |
| Central Line Associated Blood Stream Infection |
| Catheter Associated Urinary Tract Infection |
| Surgical Site Infection (colon and hysterectomy) |
| Methicillin-resistant staphylococcus aureus bacteremia |
| Clostridium Difficile Infection |

**Table 3: 2017 Healthcare Associated Complication Conditions**

**PUBLIC REPORTING OF QUALITY METRICS**

In addition to quality metrics being factored into hospital reimbursement, they are also reported publicly. The data can be accessed on Hospital Compare, https://www.medicare.gov/hospitalcompare. This gives the informed patient the ability to choose the “best” hospital and decide where they receive their care.

The data available on this site are similar to the metrics used for quality-based reimbursement adjustment. Reported measures include: patient satisfaction, timeliness of care, complications, readmissions, mortality, and cost of care among others.

**Introduction to Cost Conscious Care**

So if hospitals get a standard reimbursement based on DRG, then their “profits” are reimbursements minus the actual costs to take care of that patient. This provides a financial incentive to hospitals to appropriately utilize resources and expedite the patient’s stay in the hospital. A shorter length of stay means less cost and hopefully more profits. This is an oversimplified description since there are many other factors that influence profits.

With limited health care resources, it is important to keep costs in mind when taking care of patients. Laboratory testing, imaging, and procedures quickly add to the health care costs. As outlined in the IOM reports, we must work to reduce “waste” – costs/resource utilization that did not provide any benefit to the patient.^1^ Appropriate use of evidence based medicine and cost comparative studies should help guide us.

## How much does it cost to…?

Everyone always talks about the rising costs of health care. News reports and economics studies always use costs in the billions and trillions of dollars. That does not provide the costs for the individual patients. So I’m sure you may have ordered a few lab studies, imaging tests, and requested some procedures today. Charges for these tests vary tremendously between health care providers and regions of the country. The actual costs incurred by the hospitals for these tests are less than the charges, which are reported on the patient bill.

What do you think are the charges for each of these commonly ordered items?

| **Test** | **Estimated Charge** |
| --- | --- |
| Basic Metabolic Panel | $ |
| ABG | $ |
| CBC | $ |
| CD4 count | $ |
| ICU bed | $ |
| Isolation room | $ |
| CT Chest | $ |
| Echocardiogram | $ |
| 2 view CXR | $ |

*(Examples of estimates are shown on the next page)*

| Test | Estimated Charge |
| --- | --- |
| Basic Metabolic Panel | $100 |
| ABG | $91 |
| CBC | $76 |
| CD4 count | $280 |
| ICU bed | $2100/night |
| Isolation room | $850/night |
| CT Chest | $1200 |
| Echocardiogram | $900 |
| 2 view CXR | $158 |

Please remember that actual charges vary between hospitals and regions of the country. And we still did not include costs for medications. Physicians and patients can use websites like www.healthcarebluebook.com to estimate the costs of various services. From the costs above, you can see how medical expenses are one of the leading causes of bankruptcy.

Do we really need a BMP every day on this patient? Do we really need to repeat the CT scan when the records can be obtained from the outside hospital? Does this patient really need an isolation bed/room?

The American College of Physicians has started an initiative entitled High Value, Cost-Conscious Care (HVCCC) to “provide the best possible care to their patients and reduce unnecessary costs to the health care system.” You can learn more about this initiative and get access to resources at their website: http://www.acponline.org/clinical_information/resources/high_value_care/.

**FACILITATED EXERCISE**

Look over two of your patients that you are taking care of in the hospital currently and see what labs and other tests were likely unnecessary and list them below.

1. __________________________________________
2. __________________________________________
3. __________________________________________
4. __________________________________________
5. __________________________________________

**Conclusion**

As physicians, and especially hospitalists, it is our responsibility to improve the quality of care provided at our institutions. Through the use of the Model for Improvement and PDSA cycles we have structured mechanisms to implement change. We play a crucial role in the business of medicine at the hospital level. Our decisions are a major driver of financial outcome, quality metrics, and public perceptions for hospitals.

**References**

1. Committee on Quality Health Care in America, Institute of Medicine Crossing The Quality Chasm: a New Health System for the 21st Century. Washington, DC: National Academy Press, 2001.
2. Varkey P, Reller MK, Resar RK. Basics of quality improvement in health care. *Mayo Clin Proc*. 2007;82(6):735-739.
3. Medicare Benefit Policy Manual, CMS Pub. 100-02, Chapter 6, §20.6; same language in Medicare Claims Processing Manual, CMS Pub. 100-04, Chapter 4, §290.1.
4. ACUTE CARE HOSPITAL INPATIENT PROSPECTIVE PAYMENT SYSTEM. Centers for Medicare and Medicaid Services, Medicare Learning Network. https://www.cms.gov/Outreach-and-Education/Medicare-Learning-NetworkMLN/MLNProducts/Downloads/AcutePaymtSysfctsht.pdf Feb 2019.
5. HOSPITAL VALUE-BASED PURCHASING. Centers for Medicare and Medicaid Services, Medicare Learning Network. https://www.cms.gov/Outreach-and-Education/Medicare-Learning-NetworkMLN/MLNProducts/downloads/Hospital_VBPurchasing _Fact_ Sheet_ ICN90 7664.pdf. September 2017.
6. Hospital Readmissions Reduction Program (HRRP). Centers for Medicare and Medicaid Services. https://www.cms.gov/medicare/medicare-fee-for-servicepayment/acuteinpatientpps/readmissions-reduction-program.html.
7. Hospital-Acquired Condition Reduction Program (HACRP). Centers for Medicare and Medicaid Services. https://www.cms.gov/Medicare/Medicare-Fee-for-Service-Payment/AcuteInpatientPPS/HAC-Reduction-Program.html.
