# Supplementary material for: Implementation of a Hospital Medicine Rotation and Curriculum for Internal Medicine Residents
Source: MedEdPORTAL. 2020 Sep 29;16:10977. doi: 10.15766/mep_2374-8265.10977 (PMC7526505; doi:10.15766/mep_2374-8265.10977)
Supplement: Supplementary file 1 — RITE Orientation Email.docxPre-RITE Survey.docxPost-RITE Survey.docxModule 1 Patient Safety.docxModule 2 QI, Metrics, Reimbursement, & Care.docxModule 3 Physician Billing & Coding.docxModule 4 Transitions of Care.docx [file mep_2374-8265.10977-s001.zip › F. Module 3 Physician Billing & Coding.docx]

*Learning Objectives*

- Summarize the documentation necessary for an initial admission and subsequent daily notes
- Select the appropriate level of inpatient billing based on documentation
- Define work RVUs and describe how physicians are reimbursed

Module 3: Physician Billing and Coding

## Module 3: Physician Billing and Coding

**Pre-lecture assignment:**

Review two of your History and Physical (H&P) initial admission notes from earlier this week. Assign an initial billing code to the H&P. You can use low, moderate or high complexity as arbitrary “codes”. Just make your best guess as to what level you would bill, and we will go over it again at the end of the module. Also, you can estimate how much you think you should be reimbursed for the admission.

H&P 1 ________

H&P 2 ________

- Any websites provided in the module are optional reading

**Introduction**

The purpose of this module is to give a very basic review of inpatient physician billing. It will introduce you to the three levels of billing, documentation required for each level, and reimbursement associated with each level. Keep in mind that this module does not include observation, consultation, outpatient, ER, or critical care billing. These areas all have separate codes, levels, criteria, and reimbursement. We will begin with a clinical scenario.

**Clinical Scenario: Salary Cut?**

*After finishing residency, you go to work for a hospitalist group in BigCity, Texas. In five years, you are promoted to head of the group. During your first week on the job, the head of the hospital shows you data for your group. Work RVUs have been lower than expected over the last quarter, and unless your group reaches target RVUs, you are all facing a 20% pay cut. You think that your group may not be documenting and billing optimally.*

*The hospital administration brings you a stack of charts to review the group’s billing. As you go through the charts, you begin to wish you had learned something about billing and RVUs during your training… The first chart is as follows:*

***Initial Admission History and Physical***

Patient Name: Vera Sick

CC: nausea/vomiting

HPI: A 26-year-old female with a history of diabetes mellitus type 1 (DM1) for eight years presents with nausea and vomiting. She ran out of insulin three days ago. The next morning, she began to feel nauseous and developed crampy mid-epigastric pain that was intermittent, and 7/10 in severity. That evening, she began to have non-bloody, non-bilious emesis every time she tried to drink water or eat. She denies fevers/chills and diarrhea.

PMH: Diabetes mellitus type 1 on insulin

FH: no history of diabetes

SH: denies tobacco, alcohol, or drugs. Lives with a roommate.

ROS:

Gen: no weight loss, no fatigue

CV: no palpitations, no chest pain

Resp: no cough, no hemoptysis

GI: no diarrhea

Endocrine: positive for polyuria and polydipsia

*All other systems were reviewed and are negative.*

Physical Exam: **T** 98.8 **HR** 115 **BP** 110/76 **R** 26 O2 sat 99% on Room Air

General: thin female, tachypneic

Eyes: PERRL, no pallor, anicteric sclera

Oropharynx: dry mucous membranes, no tonsillar exudate

Cardiovascular: tachycardic, no murmurs, no carotid bruits

Respiratory: clear to auscultation bilaterally, good inspiratory effort

Abdomen: soft, mild epigastric tenderness, normal liver

Extremities: no cyanosis/clubbing, full ROM

Skin: no rashes, no masses

Neurologic: CN II-XII intact; normal reflexes

Labs

ABG: pH 7.2, pCO2 20, paO2 80

Bicarbonate 10; glucose 245; anion gap 24

UA: 3+ glucose, 4+ ketones

My review of EKG- sinus tachycardia, My review of CXR- no infiltrates

Assessment and Plan

26-year-old female with DM1 now admitted with nausea and vomiting

1. Diabetic Ketoacidosis- likely due to medication noncompliance; will continue IVF, start an insulin drip and adjust the drip per pharmacy protocol. Monitor q4h BMP and hourly accu-checks initially. Will transition to SQ insulin when the anion gap closes.

**Physician billing is important because you want to get credit and paid for the work that you do!!!**

**So how do you decide what level to bill an INITIAL INPATIENT ADMISSION?**

Each physician caring for a patient submits a bill for their professional services which is separate from hospital billing. Keep in mind that your documentation has to support the level of billing. There are 3 levels of billing and corresponding codes for each level:

Level 1 (low) 99221

Level 2 (moderate) 99222

Level 3 (high) 99223

How you decide what level to bill depends upon your documentation in 3 main categories: History, Physical Exam and Medical Decision Making (MDM). The table below summarizes the documentation needed for an initial admission note based on the 1997 Centers for Medicare and Medicaid Services (CMS) guidelines.^1^ You must fulfill the documentation in all 3 of 3 categories to bill the respective level. The text following the chart reviews each component in more detail.

|  | **Level 1 99221** | **Level 2 99222** | **Level 3 99223** |
| --- | --- | --- | --- |
| **History** | Detailed | Comprehensive | Comprehensive |
| CC  HPI  ROS  PFSH | Yes  Extended 4+  Extended 2-9  Pertinent 1 | Yes  Extended 4+  Complete 10+  Complete 3 | Yes  Extended 4+  Complete 10+  Complete 3 |
| **Physical Exam** | Detailed | Comprehensive | Comprehensive |
| Organ System (OS)  or Body Area (BA) | 2 elements in ≥ 6 OS or BA;  OR 12 elements from ≥ 2  OS or BA; OR  complete single | 2 elements from  9+ OS or BA; OR  complete single | 2 elements from  9+ OS or BA; OR  complete single |
| **MDM 2 of 3** | Low | Moderate | High |
| -No of Diagnosis  -Data Review  -Risk | Limited  Limited  Low | Multiple  Multiple  Moderate | Extensive  Extensive  High |
| Time | 30 min | 50 min | 70 min |

1. **HISTORY**

**Chief Complaint (CC)**- every note should include a chief complaint, usually stated in the patient’s own words

**History of Present Illness (HPI)**- chronological description of the signs and symptoms; four elements of the HPI OR the status of at least three chronic or inactive problems

**Review of Systems (ROS)**- inventory of body systems

Level 1- Detailed- documents at least 2 systems

Level 2-3 Comprehensive- those systems with positive or pertinent negative responses must be individually documented. For the remaining systems, a notation indicating *“all other systems are negative”* is permissible*.* In the absence of such a notation, at least ten systems must be individually documented.

These systems are considered organ systems (OS) for ROS.

*Organ Systems for ROS- Constitutional, Eyes, Ears Nose Mouth Throat (ENMT), Cardiovascular, Respiratory, GI, GU, Musculoskeletal, Skin, Neurologic, Psychiatric, Hematologic/Lymphatic, Allergy/Immunologic, Endocrine*

**PFSH (Past/Family/Social History)-**

Level 1- Detailed- document at least one item from any of the three history areas

Level 2-3- Comprehensive- document at least one item from all three history areas

1. **PHYSICAL EXAM (PE)**- the exam may include a general multisystem exam OR a single organ exam.

***Body Areas****- Head (face), Neck, Chest (breast/axillae), Genitalia/Groin/Buttocks, Back, Abdomen, Each Extremity*

***Organ Systems-*** *Constitutional, Eyes, ENMT, Cardiovascular, Respiratory, GI, GU, Hematologic/Lymphatic, Immunologic, Musculoskeletal, Neurologic, Psychiatric, Skin*

**General Multisystem Exam**

Level 1- Detailed- document at least two elements from each of six areas/systems OR at least 12 elements in two or more areas/systems

Level 2-3- Comprehensive- document at least two elements from each of nine areas/systems.

**Single Organ System Exam**

Since documentation on inpatient internal medicine typically includes a more general exam, we will focus more on the documentation necessary for the general multisystem exam instead of a single complete organ system. You can check CMS guidelines for more information on a complete single organ system/body area exam as there are variations and differing emphasis among specialties.

NOTE: You need to describe any abnormal findings of any symptomatic or asymptomatic organ systems or body areas. Just documenting “abnormal” is insufficient. It is sufficient to document “normal” for any unaffected organ systems or body areas that have normal findings.

1. **MEDICAL DECISION MAKING (MDM)**- MDM is dependent upon number of diagnoses or management options that must be considered, amount and/or complexity of data to be reviewed, and risk of complications and/or morbidity or mortality associated with the presenting problem. In this section, 2 of the 3 elements must meet or exceed the level to bill that specified level. In the 1990s, the Marshfield Clinic was a large multispecialty practice with 32 sites in Wisconsin. Medicare’s 1995 Evaluation and Management (E/M) documentation guidelines were initially tested here.^2^ The Clinic developed an audit worksheet called the “Marshfield Clinic Scoring Tool” which is now used by most physicians and coders to help with deciding the complexity of diagnosis and treatment.^3^

**• Number of Diagnosis Problem Points**

- # of self-limiting/minor problems stable/improving/worsening (max of 2) X 1 pt
- # of established diagnosis/problems (stable/improved) X 1 pt
- # of established diagnosis/problems (worsening) X 2 pts
- # of new problems with no additional work-up planned X 3 pts
- # of new problems with additional work-up planned X 4 pts

**• Number of Data points**

- Review or order lab tests X 1 pt
- Review or order radiology tests X 1 pt
- Review or order medicine tests (i.e. PFTs, cath, EKG, echo) X 1 pt
- Discuss test with performing physician X 1 pt
- Independent review of image, tracing, specimen X 2 pts
- Decision to obtain old records X 1 pt
- Review and summarize old records/history X 2 pts
- Minimal: 0-1 pt
- Limited: 2 pts
- Multiple: 3 pts
- Extensive: 4 pts

**NOTES on Data Review:**

1. If you obtain old records or further information from family/caretakers, you must elaborate in your documentation. Stating “old records reviewed” is insufficient.
2. Personal review of EKG or imaging is 2 points each, just copying interpretation of results is 1 point

**• Risk-** a more detailed table of risk can be viewed on www.cms.gov

- **Minimal**: One self-limited or minor problem
- **Low**: Two or more self-limited or minor problems, one stable chronic illness, or one acute uncomplicated illness or injury
- **Moderate**: One or more chronic illnesses with mild exacerbation, progression, or side effects of treatment, two or more stable chronic illnesses, an undiagnosed new problem with an uncertain prognosis, acute illness with systemic symptoms, acute complicated injury
- **High**: One or more chronic illnesses with severe exacerbation, progression, or side effects of treatment, acute or chronic illnesses or injuries that pose a threat to life or bodily function, an abrupt change in neurologic status

1. **BILLING ON TIME-** time may be considered the key factor in billing if >50% of face to face time is spent in counseling or coordination of care. The physician must document the total length of time and describe the counseling or activities involved to coordinate care.

**Now going back to the original H&P on Ms. Vera Sick, what level would you bill this patient?**

Let’s take it step by step—did the note include the following:

**History:**

Chief complaint:_____________________________________________

HPI:___________________________________________________________

ROS:___________________________________________________________

PFSH: ________________________________________________________

**Physical Exam:** _______________________________________________________

**Medical Decision Making:**

# of Diagnosis points: ____________________________________________

# of Data points:_________________________________________________

Risk level:___________________________________________________

Overall: ____________

Now, let’s see if there is enough documentation for the level:

Code:_______________________

The case meets a level 3--99223 billing as it contains all the necessary documentation for History, Physical Exam and MDM.

**A FEW KEY POINTS:**

**REMEMBER, the difference between level 2 and level 3 is based on Medical Decision Making, History and Physical Exam documentation required is the same!**

**REMEMBER, if there are less than 10 systems in ROS, or missing family or social history, the highest you can bill is level 1!**

**REMEMBER, avoiding overbilling is important – you should not bill a level 3 for acute cystitis just because you have enough documentation. It is much safer and more ethical to determine the level by Medical Decision Making and then make sure you have enough documentation to support that level.**

**What is the cost difference between each level of INITIAL ADMISSION billing based on 2018 Medicare Reimbursement?**

Code wRVU Reimbursement

99221 (low) 1.92 $103.70

99222 (moderate) 2.61 $139.68

99223 (high) 3.86 $207.11

**So now how do you decide what level to bill a SUBSEQUENT INPATIENT DAY?**

There are also 3 levels of billing but now history and physical exam can be more focused. You must fulfill the documentation in 2 of 3 categories to bill the respective level. 1 of the 2 must be Medical Decision Making.

|  | **Level 1 99231** | **Level 2 99232** | **Level 3 99233** |
| --- | --- | --- | --- |
| **History** | Problem Focused | Expanded Problem Focused | Detailed |
| HPI  ROS  PFSH | Brief 1-3  ___  ___ | Brief 1-3  Problem Pertinent 1  ___ | Extended 4+  Extended 2-9+  ___ |
| **Physical Exam** | Problem Focused | Expanded Problem Focused | Detailed |
| Organ System (OS)  or Body Area (BA) | 1-5 elements in 1+  OS or BA | 6+ elements in 1+  OS or BA | 2 elements in ≥ 6 OS or BA;  OR 12 elements from ≥ 2  OS or BA |
| **MDM 2 of 3** | Straightforward/Low | Moderate | High |
| -No of Diagnosis  -Data Review  -Risk | Limited  Limited  Low | Multiple  Multiple  Moderate | Extensive  Extensive  High |
| Time | 15 min | 25 min | 35 min |

**What is the cost difference between each level of SUBSEQUENT ADMISSION billing based on 2018 Medicare Reimbursement?**

Code wRVU Reimbursement

99231 0.76 $40.06

99232 1.39 $74.31

99233 2.00 $106.41

Again, keep in mind that there are different codes and documentation requirements for discharges, observation, critical care, consults, and outpatient services. The focus in this module is just to give a very basic introduction to INPATIENT physician billing.

You can see that each code corresponds to a dollar amount and wRVUs so it begs the question:

**How is physician reimbursement calculated and what are work RVUs (wRVUs)?**

Each billing code corresponds to a wRVU.

Code wRVU

99221 (low) 1.92

99222 (moderate) 2.61

99223 (high) 3.86

Medicare established a national fee schedule for physicians based on relative value units (RVUs). Total RVUs assign payment to procedures and services based on three components: physician’s wRVUs, expenses of the practice, and professional liability insurance. Specifically, wRVUs account for the time, technical skill and effort, mental effort and judgment, and stress to provide a service. Medicare adjusts payment by designating a geographic price cost index (GPCI) and pays differently for the same work depending on the practice location. Another important component is the Conversion Factor (CF), which converts the RVU into a charge and reimbursement. The calculation can be found on the CMS website. The wRVU is composed of two separate elements: time (~ 70%) and effort (~ 30%). Time is generally figured by the time required by the physician prior to a service, performing the service, and following the service or procedure such as charting. Similarly, the effort or intensity also consists of the physical effort, skill and stress involved. Increasing complexity of a medical problem equals a higher wRVU.

**So why are wRVUs important to physicians?**

wRVUs are currently the standard measurement for cost benchmarking and is used by almost all third party payers besides Medicare. They are also frequently used to determine practice efficiency and physician productivity and compensation. Average hospitalist wRVUs annually is ~***4200-4300 (2016 SHM data)***.^4^ SALARY AND BONUSES are often tied to wRVU generation!! For example, a hospitalist salary may include a base plus a bonus dependent upon annual wRVUs generated.

**Let’s Practice**

Take time to review your H&Ps from the pre-lecture assignment and discuss what level you would bill each note. Recognize the documentation you may have missed or need to elaborate upon in the future in order to bill appropriately.

______________________________________________________________________________________________________________________________________________________________________________________________________________________________________________________________________________________________________________________________________________________________________________________________________________________________________________________________________________________________________________________________**_____________________________________________________________________________________**

Take time to review some subsequent day notes and discuss the level you would bill each note.

______________________________________________________________________________

______________________________________________________________________________

______________________________________________________________________________

______________________________________________________________________________

______________________________________________________________________________

______________________________________________________________________________

______________________________________________________________________________

**Conclusion**

As a resident, you may not realize that your attending bills for their services on every patient they see with you. Keep in mind the components necessary to bill each level of service to avoid both under and over billing. Also keep in mind that it is physician billing that generates wRVUs which may impact your future salary.

**References**

1. 1997 Documentation Guidelines for Evaluation and Management Services link-https://www.cms.gov/Outreach-and-Education/Medicare-Learning-Network-MLN/MLNEdWebGuide/Downloads/97Docguidelines.pdf.
2. 1995 Documentation Guidelines for Evaluation and Management Services link- https://www.cms.gov/Outreach-and-Education/Medicare-Learning-Network-MLN/MLNEdWebGuide/Downloads/95Docguidelines.pdf.
3. Edsall RL, Moore KJ. Thinking on paper: documenting decision making. *Fam Pract Manag.* 2010;17(4):10-15.
4. Quinn, R. The State of Hospital Medicine is Strong. *The Hospitalist*. Sept 2016(9).
