# Supplementary material for: Implementation of a Hospital Medicine Rotation and Curriculum for Internal Medicine Residents
Source: MedEdPORTAL. 2020 Sep 29;16:10977. doi: 10.15766/mep_2374-8265.10977 (PMC7526505; doi:10.15766/mep_2374-8265.10977)
Supplement: Supplementary file 1 — RITE Orientation Email.docxPre-RITE Survey.docxPost-RITE Survey.docxModule 1 Patient Safety.docxModule 2 QI, Metrics, Reimbursement, & Care.docxModule 3 Physician Billing & Coding.docxModule 4 Transitions of Care.docx [file mep_2374-8265.10977-s001.zip › G. Module 4 Transitions of Care.docx]

*Learning Objectives*

- Analyze the root causes of ineffective transitions
- Review current transitions of care models
- Identify steps in the discharge process that can be improved
- Summarize the admission criteria for the different post-acute care facilities and select the appropriate discharge disposition

Module 4: Transitions of Care

**Module 4: Transitions of Care**

##

**Module 4: Transitions of Care**

**Pre-Lecture Assignment:**

Select 1-2 patients who have been readmitted within the last 30 days and review their charts to identify the reason(s) they required readmission.

**Clinical Scenario: Ms. Smith and Warfarin**

*After graduating from medical school, you decided to join a Hospital Medicine practice in AwesomeTown, Texas. The hospital administration asked that you join the Case Review committee. Since you are the “new” person, it would be rude to say no.*

*To prepare for the upcoming committee meeting, you begin reading the first chart. Ms. Smith is a 55 year-old female with a history of hypertension, diabetes mellitus, a mechanical aortic valve, and chronic kidney disease stage 5 who was admitted for new onset atrial fibrillation. Lab workup was unremarkable other than a GFR of 20 mL/min/1.73 m^2^ and echocardiogram that revealed an enlarged left atrium and diastolic dysfunction. The hospitalist started anticoagulation with warfarin. The patient was discharged on Day #3 with a prescription for warfarin and instructed to visit her PCP in 3 days for an INR check.*

*Just as you start to write “appropriate care given” on your case evaluation form, you notice a second chart. Same name but different admit date.*

*The same patient was readmitted 12 days after discharge with confusion and lethargy. A CT scan of the head showed a large intracerebral hemorrhage. Her admission INR was 8.2. She continued to do poorly despite reversal of the anticoagulation and neurosurgical intervention and died. Based on your chart review, the patient never went to her PCP appointment and never had an INR rechecked but continued to take the warfarin as prescribed. You quickly look back at the discharge prescriptions from the first hospitalization. There it is – WARFARIN 5 mg PO daily, 30 day supply, 3 refills.*

- Any websites provided in the module are optional reading

**How well do we discharge patients home?**

The case provides a clear example of an adverse event that occurred after a patient was discharged home.

Did Ms. Smith understand her discharge diagnosis and the reason for the warfarin?

Did she understand the importance of a follow up INR?

Were her discharge instructions clear?

These are just a few of many questions that need to be addressed!!

##

## INTRODUCTION

Hospital discharge is one of the most difficult times for the patient as well as the medical care team particularly if the patient is elderly. One in five hospitalizations is complicated by a post-discharge event which leads to not only an increase in emergency room visits but also in hospital readmission rates.^1-2^ Inadequate communication, poor patient understanding, and lack of follow up care are just a few of the areas that need improvement. Discharge summaries frequently lack critical data and are not sent to the primary care physicians in a timely manner. Furthermore, direct communication between physicians occurs less than 20% of the time.^3^

Hospital readmissions within 30 days is part of the Centers for Medicare and Medicaid Services (CMS) Value-Based Purchasing Program and is publicly reported. In 2009, Jencks *et al* reported that 20% of Medicare patients were readmitted within 30 days after hospital discharge.^4^ Subsequently in March 2010, President Obama signed into law a comprehensive health care reform legislation, the Patient Protection and Affordable Care Act, that contained a number of provisions that made changes to Medicare. Among these were provisions intended to reduce preventable hospital readmissions by reducing Medicare payments to certain hospitals with relatively high preventable readmissions rates. In 2012, the Hospital Readmission Reduction Program was implemented penalizing hospitals with higher-than-expected readmissions for specific diagnoses.^5^ As a result of this legislation, hospitals have been focused on implementing strategies to reduce readmissions.

Discharge planning should begin from the time of admission and continue throughout the hospitalization. Medical care team discussions that include a multidisciplinary approach should involve physicians, nurses, case managers, social workers, and pharmacists and occur on a daily basis.

**CONCEPTS IN TRANSITIONS OF CARE**

Some of the root causes of ineffective transitions of care most often described in medical literature and by experts include^6^:

• ***Communication breakdowns-*** This can include communication between physicians, physician and patient, nursing staff and patient, etc.

• ***Patient education breakdowns-*** Patients or family/friend caregivers sometimes receive conflicting recommendations, confusing medication regimens, and unclear instructions about follow-up care.

• ***Accountability breakdowns-*** In many cases, there is no physician or clinical entity that takes responsibility to assure that the patient’s health care is coordinated across various settings and among different providers.

**TRANSITIONS OF CARE MODELS**

Several evidence-based transitions of care models have been developed to improve patient outcomes. These models include the Care Transitions Intervention (CTI),^7^  Transitional Care Model (TCM),^8^  Better Outcomes for Older Adults through Safe Transitions (BOOST),^9^ The Bridge Model,^10^ Guided Care,^11^ Geriatric Resources for Assessment and Care of Elders (GRACE),^12^ and Project RED (Re-Engineered Discharge).^13^ These models include many or all of the following elements which are being researched as part of The Joint Commission enterprise transitions of care initiative:^6,14^

1. **Multidisciplinary communication, collaboration and coordination – including patient/caregiver education – from admission through transition.**

A care team – including a physician, nurse, pharmacist, social worker, and others communicates, collaborates and coordinates effectively and begins to take steps at admission and continues them through the patient’s hospital stay to assure a successful transition. Daily rounds should include educating both the patient and caregiver.

1. **Clinician involvement and shared accountability during all points of transition*.***

Both discharging and receiving clinicians are involved in and accountable for a successful transition and should be identified by name and exchange information electronically or by fax or telephone during the time of discharge.

1. **Comprehensive planning and risk assessment throughout the hospital stay.**

Each patient and caregiver has a discharge risk assessment completed usually within the first 24-48 hours of admission. Discharge planning begins immediately after admission and patients should be assessed for risk factors such as low literacy, recent admissions, chronic conditions or medications that may limit their ability to perform necessary aspects of self-care. Also, clinicians begin to assess risks that may be present at the receiving setting.

1. **Standardized transition plans, procedures and forms*.***

The following components are included in a written transition plan or discharge summary: active issues, diagnosis, medications, required services, warning signs of a worsening condition, and whom to contact 24/7 in case of emergency. Plans are

provided in the patient’s preferred language and use pictures for patients having low literacy.

1. **Standardized training*.***

Medical staff are taught the necessary steps to complete a successful transition and are engaged in real-time performance feedback. Successful transitions are made an organizational priority and performance expectation. Medical schools incorporate risk assessment, collaboration, care planning, and medication management relating to transitions of patient care into their curricula. Nursing schools and educational programs for all other health care disciplines include training on what transitions are, the risk associated with transitions, and how they can contribute to a safe patient care transition.

1. **Timely follow-up, support and coordination after the patient leaves a care setting*.***

Organizations develop a process that provides for timely post-discharge follow-up with patients. Telephone or in-person follow-up, support, and coordination by a case manager, social worker, nurse, or another health care provider 24-48 hours after discharge helps patients achieve successful recoveries. A 24/7 call center provides a recently transitioned patient or family member with information or reassurance after regular clinic hours. Having a transitional care nurse accompany the patient to the first follow-up outpatient visit can improve the health outcome, as can scheduling home care visits for the patient.

1. **If a patient is readmitted within 30 days, gain an understanding of why.**

Readmissions within 30 days of discharge can often be prevented by providing a safe and effective transition of care from the hospital to home or another setting. Convene a meeting of the care team, including the attending physician and other key staff, and the patient and family members. Ask the patient questions about what happened after discharge. Find out if there were financial or transportation barriers, and whether or not caregivers were unavailable. This important information can be used by organizations to improve care transitions for patients and family/friend caregivers.

1. **Evaluation of transitions of care measures*.***

Monitor compliance with standardized forms, tools, and methods for transitions of care. Use surveys and data collection to find root causes of ineffective transitions and to identify patient and caregiver satisfaction with transitions and their understanding of the care plan. For example, this three-item survey queries patients about key aspects of a care transition:

1. The hospital staff took my preferences and those of my family or caregiver into account in deciding *what* my health care needs would be when I left the hospital.

2. When I left the hospital, I had a good understanding of the things I was responsible for in managing my health.

3. When I left the hospital, I clearly understood the purpose for taking each of my medications.

As you can see, there are **MANY** steps that can go wrong when a patient is discharged from the hospital.

**FACILITATED EXERCISE**

1) We will now return to the case of Ms. Smith. Write down some areas that you feel could be improved in Ms. Smith’s discharge that may have prevented her adverse event and readmission based on what you now know about areas of intervention as summarized by the Joint National Commission.

Then discuss specific strategies on improving that area of discharge and how this could turn into a quality improvement project.

____________________________________________________________________________________________________________________________________________________________________________________________________________________________________________________________________________________________________________________________________________________________________________________________________________________________________

2) I’m sure you have also discharged patients that were readmitted soon thereafter. Take some time and reflect on a patient who was readmitted and create a list of changes that may have improved their transition of care home. This should include a review of the patient’s discharge instructions and summary to see if any changes (i.e. follow-up appointments, medication reconciliation) could have been made to avoid readmission.

____________________________________________________________________________________________________________________________________________________________________________________________________________________________________________________________________________________________________________________________________________________________________________________________________________________________________

3) Assemble the list of changes in order of importance to improve the discharge process for the patient above.

_____________________________________________________________________________________________________________________________________________________________________________________________________________________________________________________________________________________________________________________________________________________________________________________________________________________________________________________________________________________________________________________________________________

4) Lastly, reflect on your overall experiences (good or bad) with transitioning patients out of the hospital. There may be significant differences in your experiences depending on where you work and the ancillary staff available to help with discharge planning.

____________________________________________________________________________________________________________________________________________________________________________________________________________________________________________________________________________________________________________________________________________________________________________________________________________________________________

**Teaching Points for the Facilitated Exercise**

1) In this scenario, there were potential breakdowns in communication, patient education, and accountability. Was the patient aware of the risks of anticoagulation therapy and the necessity of close follow up? Was the post discharge appointment actually scheduled or was this the patient’s responsibility? Do we know if the receiving physician was able to see the patient in the appropriate time frame? Was there direct communication between the inpatient and receiving provider? Was there a post-discharge follow-up call by pharmacy or nursing? Was there an issue with the duration and number of refills provided by the inpatient team?

Potential QI projects: post-hospital warfarin monitoring, post-discharge telephone contacts, communication with receiving MDs, rates of post-discharge follow up

2) Appropriate discharge locations? Appropriate assistance and skilled service at home (home health aide, home health nursing, PT)? Scheduled follow-up appointments? Medication reconciliation correct and clear?

3) Scheduled appointment for INR check with plans for medication titration (with PCP or pharmacist). Direct communication with receiving provider. Post-hospital telephone visit to investigate any issues since discharge. Smaller supply of medications/refills when risk of unmonitored use is greater than benefit.

4) Difficulties with scheduling follow-up appointments. Difficulties with ensuring that patients get appropriate medications. Limited resources for at home assistance for patients. Communication barriers between physicians and patients/families.

**Now let us shift gears and review some of the facilities where patients can be discharged**

**Clinical Scenario: Where to Discharge Ms. Jones**

*Ms. Jones is a 67- year-old female with a history of stroke, ESRD on hemodialysis and a peg tube requiring bolus feedings admitted with fever and chronic back pain. She is found to have a sacral decubiti and diagnosed with osteomyelitis with MRSA from a bone biopsy. She now requires at least 6 weeks of intravenous antibiotics. Her family is unable to manage all of her health problems at home. She has Medicare. You know she requires long-term placement. Where would be the best place to discharge Ms. Jones and why?*

After short-term acute care hospitalization, about one in five of Medicare beneficiaries require continued, specialized treatment in one of the three typical Medicare Post-Acute Care settings: **IRFs, LTACHs, or SNFs.**

ACUTE CARE HOSPITAL IN THE COMMUNITY

Home/Home Health

Assisted Living

Nursing Home

POST-ACUTE CARE SETTINGS

LTACH

IRF

SNF Author Created Image

**Post-Acute Care and Community Settings and their Qualifying Criteria:**

1. Inpatient Rehabilitation Facilities (IRF)

3 Medicare Qualifying Criteria:

1. Reasonable expectation that the patient will get significant functional improvement over a reasonable amount of time
2. Patient can tolerate and participate in a minimum of 3 hours per day at least 5 days per week of Physical Therapy (PT)/Occupational Therapy (OT)/Speech/or prosthetics/orthotics; at least one of which must be PT or OT
3. Patient is medically stable
4. Long-Term Acute Care Hospitals (LTACH)
5. Acute care hospitals treat medically complex patients with prolonged length of stay (LOS)
6. Freestanding vs Hospital based
7. Medically complex, “chronic critical illness” or “post intensive care syndrome”
8. Average LOS >25 days to receive Medicare payments
9. Skilled Nursing Facilities (SNF)

- A skilled nursing facility is defined as “a nursing facility with the staff and equipment to give skilled nursing care and, in most cases, skilled rehabilitative services and other related health services.”

Medicare Qualifying Criteria for SNF:

1. 3-day inpatient hospital stay requirement
2. PT/OT/Speech one hour a day
3. Wound Care
4. PEG feedings/Tracheostomy care
5. Chronic oxygen therapy
6. IV or injectable medications
7. Assisted Living (AL)
8. Non-Medical/Self Directed Care
9. Typical Services – 3 meals a day – 24 hour security – Medication reminders – Activities of Daily Living (ADL) assistance – Transportation/Appointment assistance
10. Home Health Care- services given in home for illness and injury
11. Services include wound care, patient and caregiver education, intravenous or nutrition therapy, injections, monitoring serious illness and unstable health status

**FACILITATED EXERCISE**

## Let’s return to the clinical scenario and decide what would be the best post-discharge facility for Ms. Smith. After reviewing the facilities described above, the best place to discharge Ms. Smith at this point would be an LTACH as she requires a significant amount of medical care including long-term intravenous antibiotics, wound care for her decubiti, peg tube feedings, transport to and from dialysis, and likely PT/OT so she does not become more deconditioned.

Now review your current patient list and discuss whether you think some of the patients require placement post-discharge and which facility may be the most appropriate to meet their needs.

________________________________________________________________________________________________________________________________________________________________________________________________________________________________________________________________________________________________________________________

As you continue to manage your patients, begin to think about discharge disposition plans starting from admission as you don’t want to wait until the day of discharge to realize the patient will need placement. This will increase their length of stay sometimes by several days.

## Conclusion

As you can see from our discussion, there are many opportunities for improvement in transitions of care. Many questions remain: Can we make these transitions better for the medical team and more importantly for the patients? Can we continue to improve quality measures? The answer is yes, but it takes time and definitely requires a multidisciplinary approach. As mentioned above, these initiatives have become a priority for hospitals as reimbursements diminish especially for those patients readmitted within 30 days after discharge.

If you take the time to look around the hospital where you work, you will likely find that there are already many initiatives in progress to improve transitions of care and many areas where you can get involved.

## References

1. Forster AJ, Murff HJ, Peterson JF, Gandhi TK, Bates DW. The incidence and severity of adverse events affecting patients after discharge from the hospital. *Ann Intern Med*. 2003;138(3):161-167.
2. Forster AJ, Clark HD, Menard A, et al. Adverse events among medical patients after discharge from hospital. *CMAJ.* 2004;170(3):345-349.
3. Kripalani S, LeFevre F, Phillips CO, Williams MV, Basaviah P, Baker DW. Deficits in communication and information transfer between hospital-based and primary care physicians: implications for patient safety and continuity of care. *JAMA.* 2007; 297(8):831-841.
4. Jencks SF, Williams MV, Coleman EA. Rehospitalizations among patients in the Medicare fee-for-service program. *N Engl J Med*. 2009;360(14):1418-1428.
5. Centers for Medicare & Medicaid Services. Readmissions Reduction Program (HRRP). https://www.cms.gov/medicare/medicare-fee-for-service-payment/acuteinpatientpps/readmission-reduction-program.html.
6. Joint Commission Center for Transforming Healthcare, Improving Transitions of Care: Hand-Off Communications, http://www.centerfortransforminghealthcare.org/assets/
   1. 4/6/CTH_Handoff_commun_set_final_2010.pdf (accessed April 11, 2012).
7. Coleman EA, Parry C, Chalmers S, Min SJ. The care transitions intervention: results of a randomized controlled trial. *Arch of Intern Med* 2006;166(17):1822-1828.
8. Naylor MD, Sochalski JA. Scaling up: bringing the transitional care model into the mainstream. *Issue Brief (Commonw Fund)*. 2010 Nov;103:1-12.
9. Hansen LO, Greenwald JL, Budnitz T, et al. Project BOOST: effectiveness of a multihospital effort to reduce rehospitalization. *J Hosp Med*. 2013;8(8):421-427.
10. The Bridge Model. Illinois Transitional Care Consortium, http://www.transitionalcare.org/the-bridge-model (accessed April 11, 2012).
11. The Johns Hopkins Bloomberg School of Public Health: Guided Care, http://www.guidedcare.org (accessed April 11, 2012).
12. Counsell SR, et al. Geriatric Resources for Assessment and Care of Elders (GRACE): A new model of primary care for low-income seniors. *Journal of the American Geriatrics Society*, 2006; 54(7):1136-1141, http://www.medscape.com/viewarticle/541536 (accessed April 11, 2012).
13. Agency for Healthcare Research and Quality: Preventing avoidable readmissions: information and tools for clinicians. Project RED, http://www.ahrq.gov/qual/impptdis.htm (accessed April 11, 2012).
14. Joint Commission Resources: “*Improving Transitions of Care*,” free video.
